# A systematic review of the effectiveness of patient‐initiated follow‐up after cancer

**DOI:** 10.1002/cam4.6462

**Published:** 2023-08-21

**Authors:** Janine Dretzke, Talhah Chaudri, Rishab Balaji, Hisham Mehanna, Paul Nankivell, David J. Moore, the PETNECK2 Research Team

**Affiliations:** ^1^ Institute of Applied Health Research University of Birmingham Birmingham UK; ^2^ Birmingham Medical School University of Birmingham Birmingham UK; ^3^ Institute for Head and Neck Studies and Education University of Birmingham Birmingham UK

**Keywords:** cancer, patient‐initiated follow‐up, randomised controlled trial, systematic review

## Abstract

**Background:**

The traditional cancer follow‐up (FU) model for cancer survivors is by scheduled clinic appointments; however, this is not tailored to patient needs and is becoming unsustainable. Patient‐initiated follow‐up (PIFU) may be a more effective and flexible alternative. This systematic review aims to analyse all existing evidence from randomised controlled trials (RCTs) on the effectiveness of PIFU compared with other FU models that include routinely scheduled appointments in adults who have been treated with curative intent for any type of cancer.

**Methods:**

Standard systematic review methodology aimed at limiting bias was used for study identification, selection and data extraction. MEDLINE, Embase, CINAHL, the Cochrane Database of Systematic Reviews and Epistemonikos were searched for systematic reviews to March 2022, and Cochrane CENTRAL was searched for RCTs from 2018 (April 2023). Ongoing trial registers were searched (WHO ICTRP, ClinicalTrials.gov, April 2023). Eligible studies were randomised controlled trials comparing PIFU with an alternative FU model in adult cancer survivors. Risk of bias assessment was via the Cochrane risk of bias tool‐2. Meta‐analysis was precluded by clinical heterogeneity and results were reported narratively.

**Results:**

Ten RCTs were included (six breast, two colorectal, one endometrial cancer and one melanoma, total *n* = 1754); all studies had risk of bias concerns, particularly relating to how missing data were handled, and populations were unlikely to be representative. Limited findings in breast cancer suggested that type of FU does not affect recurrence detection or patient‐related outcomes, while PIFU may reduce the number of clinic visits. Adding patient‐led surveillance to routine FU may increase melanoma detection. Evidence for other types of cancer is too limited to draw firm conclusions.

**Conclusions:**

PIFU may be a viable FU model in breast cancer, but further research is needed for other types of cancer and on long‐term outcomes. A protocol was registered with PROSPERO (CRD42020181424).

## 1 BACKGROUND

The number of cancer survivors is rising worldwide, with approximately 43.8 million cancer survivors in 2018.^1^ Most of these will receive long‐term follow‐up (FU), which traditionally involves scheduled outpatient visits in a hospital setting.^2^ Current models of FU in cancer are seen as unsustainable by health care providers, both financially, and practically in terms of managing increased pressures on outpatient systems.^3^, ^4^ Current models are also seen as inflexible in terms of meeting patient needs and this has led to calls for alternative models of FU to meet patient needs in a more tailored and flexible way and to improve cost‐effectiveness.^3^, ^4^, ^5^ An alternative FU model is patient‐initiated FU (PIFU). This generally involves patients triggering FU appointments according to their individual needs and symptoms (‘on‐demand’) with subsequent rapid access to specialist care, while routine clinic appointments are no longer, or less frequently, scheduled. Rapid access to appointments can be difficult where clinics are primarily dedicated to scheduled FU, so any reduction in such appointments may free up availability for ‘on‐demand’ appointments.^6^ Patients are provided with information on signs and symptoms of recurrence to help them decide when to initiate contact with a designated health care professional (such as a specialist nurse). Despite foregoing regular outpatient visits, some models of PIFU include regular imaging, for example in breast^7^ and colorectal cancer.^8^

There is uncertainty however around the effectiveness and cost‐effectiveness of PIFU. It is thought that PIFU may reduce diagnostic delay by enabling more rapid access to specialist care when needed.^6^ Foregoing scheduled visits (and potentially missed appointments) is likely to reduce costs and avoid unnecessary visits by patients who are potentially well.^3^ It is known that FU visits after cancer cause anxiety to patients, though some patients find face‐to‐face FU appointments with a doctor reassuring.^9^, ^10^ On the other hand, there may also be risks associated with PIFU in terms of potentially missed symptoms or recurrences, as it relies on patients' willingness and ability to self‐manage and initiate contact.^3^, ^11^

Many studies have explored alternative models in both cancer and other chronic conditions, including nurse‐led FU, GP‐led FU, models of shared FU across primary and secondary care, different frequencies of FU and patient‐initiated FU (PIFU). Several systematic reviews exist of such studies, for example focussing on chronic conditions^3^, ^5^ or cancer.^2^, ^6^, ^12^, ^13^ One of these systematic reviews had a similar aim to ours but includes fewer RCTs.^13^ This systematic review aims to analyse all the existing evidence from randomised controlled trials (RCTs) on the effectiveness of PIFU compared with FU models that include routinely scheduled appointments in adults who have been treated with curative intent for any type of cancer. Any effectiveness outcomes have been considered including mortality, recurrence, quality of life, treatment adherence, contact with health professionals and patient satisfaction.

## 2 METHODS

### 2.1 Searches

Randomised controlled trials were identified through checking the included studies in existing systematic reviews with the reviews identified through searching MEDLINE, Embase, CINAHL, the Cochrane Database of Systematic Reviews and Epistemonikos from inception to March 2022. This was supplemented with searches for more recent RCTs in Cochrane CENTRAL (2018 to April 2023). Ongoing trial registers were searched (WHO ICTRP, ClinicalTrials.gov). There was no restriction by language of publication. Searches combined text and index terms (where implemented) relating to PIFU and cancer combined with systematic review filters (where needed); as the terminology used for PIFU is variable, several alternate terms were used to ensure comprehensiveness (see supplemental material for search strategies).

### 2.2 Study eligibility criteria and screening

Two reviewers independently screened titles and abstracts or full texts where necessary, using pre‐defined inclusion criteria. Disagreements were resolved through discussion and reasons for exclusion were noted. Rayyan software (http://rayyan.qcri.org, Qatar Foundation, Qatar) was used for screening. Randomised controlled trials were eligible where they compared PIFU with another type of FU in adult cancer survivors who have completed curatively intended cancer treatment. In the PIFU arm, PIFU alone could be the model of FU, or it could be an add‐on to routine care (either with a reduced or standard number of pre‐scheduled appointments). Eligible studies could therefore be those evaluating PIFU versus routine care; PIFU + routine care versus routine care; or PIFU versus PIFU + routine care. Either arm could receive cancer specific imaging or other diagnostic tests. Any type of cancer and any effectiveness outcomes at any time‐point were eligible.

### 2.3 Data extraction and risk of bias assessment

All systematic reviews were checked for relevant RCTs and full texts of these, and any additional RCTs, obtained. Data were extracted by one reviewer using a piloted data extraction form and checked by a second. Disagreements were resolved through discussion. Data were extracted on population characteristics, type of PIFU and routine FU, type of outcome and findings. The Cochrane risk of bias tool‐2 was used to assess the risk of bias.^14^ The effect of assignment to intervention (the ‘intention‐to‐treat’ effect) was assessed for all studies (rather than the ‘per protocol’ effect). The majority of studies used several questionnaire‐based outcomes, and risk of bias assessment was undertaken for the primary outcome (where specified in each study) as this was deemed to be representative of the overall methodology across all outcomes. The risk of bias was not additionally assessed for more objective outcomes within the same study, such as recurrence or mortality, unless this was the only outcome assessed.

### 2.4 Synthesis

Findings were grouped by type of cancer (breast, endometrial, colorectal, melanoma) and described on an outcome‐by‐outcome basis. Pooling of findings for any of the outcomes was precluded by clinical and/or methodological heterogeneity. Formal assessment of small study effects bias (e.g. though funnel plots) was therefore also not possible. RCTs varied in outcomes assessed, types of questionnaires used, outcome metrics used (mean, median or %), the presentation of summary or sub‐scale scores (for questionnaires), length of FU and the point in their care where patients were allocated to different FU strategies.

## 3 RESULTS

**FIGURE 1:**
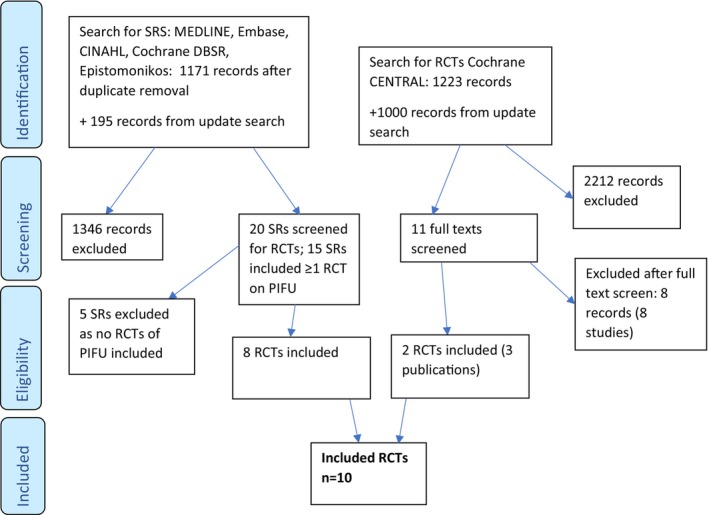
PRISMA flowchart.

Ten RCTs were included in total. Eight were identified across 15 systematic reviews that included at least one RCT of PIFU compared with another FU model,^2^, ^3^, ^5^, ^6^, ^12^, ^15^, ^16^, ^17^, ^18^, ^19^, ^20^, ^21^, ^22^ and two from the supplementary searches (Figure 1 PRISMA flowchart). This included six RCTs in breast cancer,^7^, ^23^, ^24^, ^25^, ^26^, ^27^ two in colorectal cancer reported in three publications,^8^, ^28^, ^29^ one in endometrial cancer^30^ and one in melanoma^31^ (see Supplementary material for excluded studies).

### 3.1 Risk of bias assessment

The main risk of bias found was a lack of blinding in all studies, which is unavoidable due to the nature of the intervention (supplemental material for risk of bias assessment). This is more relevant for subjective outcomes (e.g. patient completed questionnaires) where knowledge of the intervention may bias responses, and less relevant for more objective outcomes such as recurrence. There were concerns, or a lack of information, around how missing data were handled in eight RCTs^8^, ^23^, ^24^, ^25^, ^26^, ^27^, ^28^, ^31^ and there was a lack of information on potential differences between patients who did, or did not, contribute to analyses in eight RCTs.^7^, ^8^, ^23^, ^24^, ^25^, ^26^, ^27^, ^28^, ^31^ There were some concerns relating to risk of bias arising from randomisation in five RCTs,^8^, ^24^, ^25^, ^27^, ^28^ and some concerns relating to selection of reported results in six RCTs.^7^, ^23^, ^24^, ^25^, ^26^, ^28^ Some risk of bias concerns may have arisen due to a lack of reporting and the risk of bias ratings should therefore be seen as indicative of potential issues rather than as evidence of risk of bias. We assessed risk of bias based only on one selected outcome for each study; for 9 out of 10 studies this was a questionnaire‐based, patient‐reported outcome, but for the remaining study^28^ this was a more objective outcome (tumour recurrence) as no questionnaire‐based outcomes were reported. This is likely to have led to slightly lower risk of bias ratings for this study for some of the domains.

### 3.2 Breast cancer

Of the six RCTs in breast cancer, four were from the UK,^23^, ^24^, ^25^, ^26^ one from Sweden^7^ and one from Denmark^27^ (*n* = 61 to 264), Table 1 for main study characteristics). There was variability between RCTs in the stage of breast cancer included, timing of PIFU initiation in the treatment pathway, length of FU (1–5 years) and recruitment rates (between 50% and 93% of those eligible). There was variability in which characteristics were reported for participants and non‐participants, and differences were not always consistent between studies; non‐participants were, younger, with a more recent diagnosis and a higher stage of primary disease (*p*‐values not reported)^24^; older and with lower general health questionnaire scores for psychosocial morbidity in the proportion that this was measured in (*p* = 0.02)^26^; had a lack of technical skills/access to computer, had co‐psychosocial comorbidities or cognitive impairment or declined due to personal reasons (statistically significant differences between participants and non‐participants not reported)^27^; or were not significantly different in age to participants.^23^ Four RCTs compared PIFU with routine FU, and two^24^, ^25^ compared PIFU with routine FU + PIFU. All studies had on‐demand access to a nurse or clinic in the PIFU arms, scheduled clinic visits in the routine FU arm and regularly scheduled mammograms in both PIFU and routine FU arms. Additional components were sometimes offered to one or both treatment arms, including a patient self‐management programme, instructions for monthly self‐examination, collection of ePROs which in turn could trigger contacts or a clinic review at the end of the study (Table 2 components of PIFU).

**TABLE 1:** Main study characteristics RCTs.

Author, year, country	Cancer stage, treatment/FU stage at recruitment	Recruitment/eligibility criteria	*n*, mean age (SD), sex	Description of PIFU	Description of control (routine FU)	Length of FU	Outcomes assessed
Brown 2002, UK^23^	Stage I breast cancer, treatment at least 1 year before recruitment	From four clinics, which were similar in way of delivering FU. Eligibility: Patients had to have treatment at least 1 year before recruitment, with no signs of recurrence. Patients deemed not suitable by medical staff or BCNs were excluded (common reasons anxiety and current personal problems). Recruitment rate: 50% from those eligible. Differences between participants/non‐participants: no statistically significant difference in age (no further details reported)	*n* = 61, age 68 (range 53–87) PIFU, age 63 (range 48–83) control, all female	No routine clinic appointments. Written information on the signs and symptoms of recurrence. Advised to contact BCN by telephone if any Problem. Yearly mammogram	Routine clinic FU with examinations by doctor. Opportunity to ask questions at clinic. Yearly mammogram	1 year	Hospital Anxiety and Depression Scale; EORTC Quality of Life QLQ‐C30; EORTC QLQ BR23; contact with HCPs
Gulliford 1997, UK^24^	Any stage breast cancer, most stage I or II; years since diagnosis between <2 and >5.	All patients seen at single centre over 24 months. Eligibility: lack of known recurrence of cancer; current lack of symptoms consistent with recurrence; no active management apart from adjuvant tamoxifen; home telephone; fluency in English. Recruitment rate: 93% of those eligible opted for randomisation. Differences between participants/non‐participants: those declining had trend towards higher stage of primary disease, younger age, more recent diagnosis (not reported whether statistically significant)	*n* = 196, age ≤ 49: 30% PIFU, 28% control age 50–65: 51% PIFU, 49% control age > 65: 20% PIFU, 23% control	Routine mammograms scheduled (frequency dependent on treatment). All instructed to self‐examine monthly and telephone if symptom development or other concerns		16 months (median)	Contact with HCPs/resource use, patient preferences for FU. Questionnaires based on the Medical Research Council quality of life questionnaire (NB No QoL data presented: ‘*The present study is limited by its size and duration and does not justify publication of immature data concerning recurrence and quality of life’.)*
				Review/clinic visit only at time of mammogram	Routine schedule of clinic visits		
Kirshbaum 2017, Australia & UK (UK setting)^25^	Stage I or II breast cancer; patients post‐surgery and where possible pre‐radiotherapy	Recruitment by BCN from single centre. Eligibility: Stage I or II and clinically at low risk of recurrence; exclusion: stage III or IV breast cancer; received adjuvant chemotherapy; increased risk factors (young age, significant family history or bilateral cancers). Recruitment rate: no details. Differences between participants/non‐participants: no details; demographic, social and comorbidity factors not recorded on the participants	*n* = 112, 60.7 (10.86) PIFU, 60.5 (9.79) control, all female	All: psycho‐educational self‐management programme (1/2 day sessions over 4 weeks) before randomisation.		5 years	Hospital Anxiety and Depression Scale; EORTC Quality of Life QLQ‐C30; EORTC QLQ‐BR23
				No routine FU. Resource pack including details on how to access breast surgical services if any concerns, via telephone helpline run by BCN. Nurses can instigate investigations and complete breast examinations and biopsies. Yearly mammogram for 5 years	Routine hospital aftercare. Yearly mammogram for 5 years.		
Koinberg 2004, Sweden^7^	Stage I or II breast cancer, patients newly diagnosed	Consecutively selected patients at three centres. Eligibility: newly diagnosed Stage I or II breast cancer. Recruitment rate: no details. Differences between participants/non‐participants: no details *NB One centre excluded during study as study arms were deemed to be too similar (135/400 patients)*	*n* = 264, 60.0 (10.3) PIFU, 58.8 (10.1) control, all female	Meeting with nurse 3 months after treatment. Information provided on how to recognise recurrence. Patient asked to contact nurse as soon as she had any questions or symptoms that could be related to breast cancer. Nurse could consult physician if needed and had rapid access to specialists. Yearly mammogram for 3 years, then referral back to routine screening	4 clinical examinations per year (first 2 years), bi‐annual examinations up to 5 years, yearly after 5 years. Yearly mammogram. Blood tests, chest x‐ray or other imaging on clinical indication	5 years	Hospital anxiety and depression scale; satisfaction and accessibility scale; contact with HCPs; recurrence; mortality
Sheppard 2009, UK^26^	Any stage breast cancer, patients at two‐year clinical review	Recruitment from specialist breast unit. Eligibility: patients diagnosed 2 years prior, no clinical signs of recurrence and not undergoing current treatment except endocrine treatment only. Recruitment rate: 72% Differences between participants/non‐participants: non‐participants were older than participants and had lower GHQ12 scores (measuring psychological morbidity, *p* = 0.02; NB the latter only measured in a proportion of non‐participants)	*n* = 237, 57 (11) PIFU, 58 (10.7) control, all female	No routine appointments. Information on how to contact the BCN if concerned. BCNs underwent training in clinical examination, physical assessment and subsequent management of symptoms before study. Yearly mammogram. At the end of the study period all participants in the PIFU group were invited for clinical review to check recurrences had not gone unreported	FU appointments for clinical review recurring every 6 months. Yearly mammogram.	18 months	General Health Questionnaire (GHQ12); Functional Assessment of Cancer Therapy (FACT) questionnaire with the addition of the breast and endocrine subscales (FACT‐B/ES); fear of recurrence; isolation; contact with HCPs; number of recurrences detected
Riis 2020, Denmark^27^	Hormone‐receptor Positive, early stage breast cancer (Stage I–III), participation in the study within 9 months of initiation of endocrine therapy	Recruitment from one centre. Eligibility: Danish‐speaking patients, age ≥ 50, post‐menopausal at time of diagnosis, hormone‐receptor positive breast cancer (Stage I–III), classified as in complete disease remission after primary surgery, and scheduled for 5+ years of adjuvant endocrine therapy. Recruitment: 65% of eligible patients. Differences between participants/non‐participants: 35% eligible patients excluded due to lack of technical skills/access to computer, co‐psychosocial comorbidities or cognitive impairment, declined due to personal reasons or were not assessed. Statistically significant differences between participants and non‐participants were not reported	*n* = 129, 64.4 (no SD) PIFU, 64.2 (no SD) control, all female	All: encouraged to use their GP or counselling centres hosted by the Danish Cancer Society; referral to other hospital departments or oncological rehabilitation specialists if indicated.		2 years	Patient Experience Questionnaire (PEQ); use of consultations; adherence to treatment; EORTC QLQ‐C30; EORTC breast cancer module (QLQ‐BR23
				No routine consultations (apart from administration of medications). Patient could request additional consultation or phone call with a specialised nurse (via e‐questionnaire, email or telephone call). Consultation planned according to urgency of the reported problems. ePROs collected every 3 months; if a patient reported severe side effects/emerging symptoms, but did not request a consultation, she was contacted by email to discuss how to handle these problems as part of individualised care package. No details on mammogram in this group, but assume provided	Pre‐scheduled consultations at 6 monthly intervals for a period of five years; examination by clinician. Mammogram and US 18–24 months after surgery, then every 2 years		
Jeppesen 2018, Denmark^30^	Endometrial cancer stage I or II, randomisation at time of staging after surgery	Recruitment from four centres. Eligibility: patients treated with curative intent for stage I or II endometrial cancer; exclusion criteria were treatment with adjuvant chemotherapy or radiation therapy, participation in a project with FU examinations or FU for other gynaecological malignancy, tumours with high‐risk histology and inability to complete questionnaires, because of mental impairment or insufficient literacy in Danish. Recruitment rate: 70% of those eligible randomised. Differences between participants/non‐participants: more nonparticipants had cardiovascular disease compared with participants (73% vs. 54%, *p* = 0.03)	*n* = 212; 63.4 (8.3) PIFU, 66.5 (8.9) control, all female	No scheduled examinations. Information given verbally on alarm symptoms that required examination. Self‐referral via telephone number of a designated project nurse at the department of gynaecology or, if preferred, GP contact	Routine FU, in accordance with Danish guidelines. 3‐year FU period, with scheduled visits every 4–6 months in the first 2 years and every 6 months during the third year. FU visits included clinical and gynaecological examinations with vaginal ultrasound, biopsies in case of suspicious findings and imaging in case of symptoms or histologically verified recurrence	10 months	Fear of Cancer Recurrence Inventory (FCRI); contact with HCPs
Hovdenak Jacobsen 2021, Denmark^8^, ^29^	Rectal cancer (Stage I–III), after completed treatment.	Patients were recruited from four surgical centres covering one third of the Danish population. Eligibility: patients who underwent major surgical resection with free resection margins for primary UICC Stage < IV rectal adenocarcinoma (ICD‐0‐C20.9), age ≥ 18 and fluent in Danish. Excluded: patients diagnosed with metastases, synchronous cancer, cognitive deficit, and life expectancy < 2 years as assessed by the surgeon, participation in competing follow‐up studies and insufficient mastery of the Danish language. Recruitment rate: 66% 0f those eligible Differences between participants/non‐participants: more female, older patients and those with a poorer performance status at the time of diagnosis among non‐participants (statistically significant difference)	*n* = 336, 65.2 (8.0) PIFU, 65.6 (9.9) control, 67% male	Standardised education and thorough patient information regarding relevant symptoms; patient access to unrestricted self‐referral to a dedicated nurse. Management of any problem reported was based on standardised response algorithms. No planned clinical visits except from the initial patient education. CT (+ CEA) assessed at 12 months and scheduled for 36 month. Patients were informed of the computerised tomography (CT) scan results by mail or telephone, unless clinical assessment was specifically indicated	Routine clinical doctor visits (with rectospcopy), pre‐scheduled at 6‐, 12‐, 18‐, 24‐ and 36‐ months. CT (+CEA) at 12 and 36 months	1 year	Hospital contacts, outpatient doctor visits, outpatient nurse visits, telephone and mail contacts, patient involvement, patient satisfaction
Ohlsson 1995, Sweden^28^	Patients undergoing resection with curative intent for colorectal cancer (Dukes stage A, B or C). Randomised 3 months after surgery.	Two surgical centres. Eligibility: patients undergoing resection with curative intent for colorectal cancer; exclusion: patients operated with local excision or having demonstrable distant metastases, patients in whom age or severe illness was considered to preclude treatment of recurrent disease, inability to cooperate, ulcerative colitis, Crohn's disease, familial polyposis, and incomplete colonoscopy together with uncertain findings at the barium enema examination. Recruitment rate: no details. Differences between participants/non‐participants: no details	*n* = 107, 65.5 (45.7–83.6) PIFU, 65.7 (40.6–83.3) control, 48% male	No planned FU visits. Written instruction on leaving faecal samples with district nurse for examination of haemoglobin every third month during the two first years after surgery and then once a year. Instructions to contact surgical department if experienced any problems with colostomy, abdominal or perineal pain, altered bowel movements, change in faecal colour, micturition problems or weight loss	‘Active’ FU with schedule of examinations/tests of over 5 years (includes, at pre‐specified intervals, physical examinations proctosigmoidoscopy, blood tests, chest x‐rays, colonoscopy, CT of pelvis)	5.5 to 8.8 years (median 6.8)	Tumour recurrence, median time to detection of recurrence, re‐resection, survival, 5‐year survival, cancer‐specific 5‐year survival, 5‐year survival after recurrence, median survival after recurrence, adherence to FU programme
Ackermann 2022, Australia^31^	Patients treated for a Stage 0, I or II localised melanoma; no restrictions on time since diagnosis and treatment of first primary melanoma	Nice physicians recruited patients from two melanoma specialty clinics and one primary care skin cancer clinic in New South Wales. Eligibility: patients treated for a Stage 0, I or II localised melanoma who were attending routinely scheduled clinics, owned a compatible smartphone, were able to perform skin self‐examination (SSE, as determined by the recruiting physician), had a skin‐check partner (i.e. a family member or friend) who could assist them, were able to understand English, and had no history of cognitive impairment. Recruitment rate: 31% of eligible patients. Differences between participants/non‐participants: ‘*clinical and demographic characteristics of the eligible and contacted and the patients randomised were similar’*. (*p*‐values not reported)	*n* = 100, 58.7 (12.0), 54% male	Patient‐led surveillance was composed of instructional videos on how to perform SSE, reminders to undertake SSE, a mobile dermatoscope attached to their smartphone, an app that facilitated store‐and‐forward teledermatology, and fast‐tracked unscheduled clinic visits	Both groups received usual care: routinely scheduled or unscheduled clinic visits as determined by the treating physician(s) and an educational booklet, which included instructions on SSE and what signs to look for that might indicate a possible melanoma	6 months	Primary outcome: proportion of eligible and contacted patients who were randomised. Secondary outcomes: patient‐reported outcomes (SSE knowledge, attitudes, and practices, psychological outcomes, other health care use) and clinical outcomes (clinic visits, skin surgeries, subsequent new primary or recurrent melanoma)

**TABLE 2:** Components of PIFU and routine FU.

Study	Type of FU	Patient information or education	Contact nurse/clinic if problem/symptom; on demand access to clinics	Regularly scheduled scans^a^	Regularly scheduled clinics	Other components
Riis 2020^27^ *Breast cancer*	PIFU		✓	✓		ePROs collected every 3 months, could trigger contact from clinic to patient Advised to use GP or counselling centres; referral to other specialists if indicated
	Routine FU			✓	✓	Advised to use GP or counselling centres; referral to other specialists if indicated
Kirshbaum 2017^25^ *Breast cancer*	PIFU	✓	✓	✓		Psycho‐educational self‐management programme
	PIFU + Routine FU	✓	✓	✓	✓	Psycho‐educational self‐management programme
Sheppard 2009^26^ *Breast cancer*	PIFU		✓	✓		Clinical review at end of study to check for missed recurrences
	Routine FU			✓	✓	
Koinberg 2004^7^ *Breast cancer*	PIFU	✓	✓	✓		
	Routine FU			✓	✓	
Brown 2002^23^ *Breast cancer*	PIFU	✓	✓	✓		
	Routine FU			✓	✓	
Gulliford 1997^24^ *Breast cancer*	PIFU		✓	✓		Instruction to self‐examine monthly Clinic only at time of mammogram
	PIFU + Routine FU		✓	✓	✓	Instruction to self‐examine monthly
Jeppesen 2018^30^ *Endometrial cancer*	PIFU	✓	✓			
	Routine FU			✓	✓	
Ohlsson 1995^28^ *Colorectal cancer*	PIFU		✓			Information on symptoms to look out for provided Written instructions to leave faecal samples with district nurse at regular intervals
	Routine FU			✓	✓	
Hovdenak Jacobsen 2021^8^, ^29^ *Rectal cancer*	PIFU	✓	✓	✓		
	Routine FU			✓	✓	Prescheduled outpatient rectoscopies
Ackermann 2022^31^ *Melanoma*	PIFU + routine FU	✓	✓		✓	Reminders to perform skin self‐examination, patient‐performed dermoscopy, teledermatologist assessment; booklet on melanoma; instructional video on how to perform skin self‐examination
	Routine FU	✓			✓	Booklet on melanoma

^a^ All breast cancer studies included regular mammograms; Ohlson 1995: routine FU arm included examinations, proctosigmoidoscopy, blood tests including carcinoembryonic antigen (CEA), chest x‐rays, colonoscopy, pelvic CT; Hovdenak Jacobsen 2021: both arms had scheduled CT scans (+CEA test) at 1 and 3 years; Jeppesen 2018: routine clinic visits included clinical and gynaecological examinations with vaginal ultrasound.

#### 3.2.1 Recurrence and mortality

There were no significant differences in reported recurrence (based on three studies,^7^, ^23^, ^26^ see Supplementary material for details of all results), or time to recurrence or all cause death (based on one study^7^). Two studies reported how recurrences were detected: in the PIFU arms this was by GP referral (*n* = 2)^23^; or emergency admission (*n* = 1), after mammogram (*n* = 1), GP emergency admission (*n* = 1), or after contact with BCN and bone scan (*n* = 2).^26^ In the routine FU arms, recurrences were detected via a clinic visit (*n* = 1) or via BCN contact and GP referral (*n* = 1)^23^; or via emergency admission (*n* = 2), GP emergency admission (*n* = 1), or patient‐identified, but waited until next scheduled FU, *n* = 1.^26^

#### 3.2.2 Quality of life, psychological morbidity and fear of recurrence

Based on four studies,^23^, ^25^, ^26^, ^27^ there were no significant differences in the EORTC QLQ‐C30, EORTEC QLQ‐BR23 or the FACT‐G questionnaires, except for the arm and breast symptom sub‐scale scores of the EORTEC QLQ‐BR23 (in favour of routine FU).^23^ No significant differences were found in anxiety and depression (based on three studies^7^, ^23^, ^25^), in psychological morbidity (based on the GHQ12 questionnaire^26^) or fear of recurrence and levels of isolation (based on one study^26^).

#### 3.2.3 Patient satisfaction and preferences

Two studies^7^, ^27^ found similar levels of satisfaction with accessibility by phone, accessibility to medical centre and satisfaction with medical centre (Satisfaction and Accessibility Scale), similar proportions of (dis‐)satisfied patients in both groups and similar proportions of unmet needs in both groups (based on the Patient Experience Questionnaire). One study^24^ (comparing PIFU with routine FU + PIFU) found similarly high proportions in both arms found clinic visits reassuring and wished to continue with routine hospital‐based FU, though more patients in both arms preferred a less frequent schedule. In the latter study, patients in the PIFU arm still had a review/clinic visit at the time of mammogram.

#### 3.2.4 Contact with health care professionals

One study reported significantly more clinician consultations overall^27^ and another significantly more physician visits in the routine FU arm^7^; in the latter study there were significantly more nurse visits in the PIFU arm. Two studies reported similar levels of contacts with a (specialist) nurse or via telephone compared with routine FU.^23^, ^26^ Referrals to hospital by a GP were similar in one study.^23^ In the study comparing PIFU with routine FU + PIFU there were similar numbers of phone calls and (cancer and non‐cancer related) GP visits.^24^ One study reported a significantly greater number of mammograms in the PIFU arm and also more pulmonary x‐rays (though the difference for the latter was not statistically significant); other imaging or laboratory investigations were similar.^7^

#### 3.2.5 Treatment adherence

One study reported adherence to treatment.^27^ Patient adherence to endocrine treatment and proportion of patients changing their endocrine treatment were similar in routine FU and PIFU arms.

### 3.3 Colorectal cancer

Two studies were included (Sweden, *n* = 107^28^ and Denmark, *n* = 336^8^, ^29^). In the Swedish RCT, patients in the PIFU arm were instructed to contact the surgical department if they had any symptoms and it was also recommended they leave faecal samples with the district nurse at regular intervals.^28^ The routine FU arm had scheduled clinic visits with a range of investigations (including physical examinations, proctosigmoidoscopy, blood tests including carcinoembryonic antigen (CEA), chest x‐rays, colonoscopy, pelvic CT). Median follow‐up was 6.8 years. The recruitment rate was 66% in one study, and non‐participants were more likely to be female, older and with a poorer performance status at the time of diagnosis (statistically significant differences).^8^ There were no details on recruitment rate or on differences between participants and non‐participants in the other study.^28^ In the Danish pilot RCT PIFU was based on patient education on symptoms and access to unrestricted self‐referral to a dedicated nurse, while the routine FU arm had no option for self‐referral but received prescheduled outpatient rectoscopies.^8^ CT scans (+ CEA test) were scheduled in both arms at 1 and 3 years. There was a non‐participation rate of 34%; non‐participants were mainly female, significantly older and those with poorer performance status at time of diagnosis. Follow‐up was 1 year.

#### 3.3.1 Recurrence and mortality

No statistically significant differences were found for recurrence‐ and survival‐related outcomes based on one study, though they were all slightly less favourable for the PIFU arm (41% (PIFU) vs. 28% (control) mortality; 67% vs. 75% five‐year survival; 2.0 vs. 1.7 years median time to first recurrence; 2.7 vs. 3.5 years median survival after recurrence).^28^ Four of five re‐resections after recurrence were in patients with asymptomatic recurrence in the routine FU arm.

#### 3.3.2 Contact with heath care professionals

One study reported significantly fewer outpatient visits to a doctor in the PIFU arm as per the design of the trial.^8^ There were no significant differences in extra clinical visits (not routine), visits initiated by patients, nurse visits or telephone consultations, though a significantly greater proportion of patients (17% vs. 7%) had ≥15 visits in the PIFU arm.

#### 3.3.3 Adherence to PIFU or routine FU protocols

In one study a high proportion in the PIFU arm (38%) had planned routine clinic visits due to non‐compliance with the PIFU protocol by the hospital and/or because it was deemed clinically necessary (i.e. for post‐operative complications).^8^ In the other study 35% of patients in the PIFU arm did not have any contact at all after randomisation, whereas there was 98% compliance in the routine FU arm.^28^

#### 3.3.4 Patient satisfaction and quality of life

Patient involvement and satisfaction score were significantly higher in the PIFU arm for 2/6 (involvement) and 5/5 (satisfaction) items, respectively, based on one study.^8^ In the same study there were no statistically significant differences between groups based on Functional Assessment of Cancer Therapy—colorectal (FACT‐C) and FACT‐C/Treatment Outcome Index (TOI).^29^

### 3.4 Endometrial cancer

One Danish RCT (*n* = 212) was identified in patients treated with curative intent for Stage I or II endometrial cancer.^30^ In the PIFU arm, patients received no scheduled examinations, but were given information on alarm symptoms and told to self‐refer by telephoning a designated project nurse or GP if preferred. The control arm received routine FU care with regular clinic visits which included clinical and gynaecological examinations with vaginal ultrasound. Recruitment rate was 70% with more non‐participants having cardiovascular disease compared with participants (*p* = 0.03). Follow‐up was 10 months. Survival‐ or recurrence‐related outcomes were not assessed.

#### 3.4.1 Fear of cancer recurrence

There was a statistically significant greater reduction in fear of cancer (measured by the Fear of Cancer Recurrence Inventory) in the routine FU group compared with the PIFU group. There was no difference in the proportion of patients with ‘clinical’ FCR, as defined by a cut‐off on the severity subscale of the FCRI.

#### 3.4.2 Contact with health professionals

There were significantly fewer examinations performed at the hospital in the PIFU group, but no differences for telephone contacts with the hospital, GP visits or visits to private practice gynaecologists.

### 3.5 Melanoma

One pilot RCT from Australia was identified (*n* = 100).^31^ Patient‐led surveillance consisted of usual care plus reminders to perform skin self‐examination, patient‐performed dermoscopy, teledermatologist assessment and fast‐tracked unscheduled clinic visits. Clinician‐led surveillance consisted of usual care. Patients in both arms had scheduled visits (as per treating clinician) so the PIFU element was an add‐on for one arm. Eligibility criteria included owning a compatible smartphone and having a skin check partner. Recruitment rate from eligible patients was 31%; main reasons for non‐participation included the patient declining, not being contacted or an unclear reason. Participants and non‐participants were similar in terms of clinical and demographic characteristics (*p*‐values not reported). Follow‐up was 6 months.

#### 3.5.1 Recurrence

There were more diagnoses of recurrent or a new melanoma in the patient‐led surveillance group (RR 10, 95% CI −2, 23) though the difference was not statistically significant, and a similar number of keratinocyte cancer diagnoses.

#### 3.5.2 Other outcomes

Patient‐led surveillance generally increased level of confidence and knowledge of SSE as well as positive beliefs around SSE, though not always significantly. It also increased frequency of SSE and likelihood of examining all body areas. There were no significant differences in fear of recurrence or new melanoma, or depression and anxiety. There was a significant increase in clinic visits with patient‐led surveillance, but an equal likelihood of having surgically lesions excised in both groups.

## 4 DISCUSSION

Six RCTs contributed evidence on follow‐up models in breast cancer.^7^, ^23^, ^24^, ^25^, ^26^, ^27^ Findings suggested that PIFU does not reduce recurrence detection, lower quality of life, increase psychological morbidity or patient dissatisfaction, but may reduce the number of consultations with a clinician while having little effect on nurse or telephone contacts. However, some evidence also suggests an increase in nurse consultations and mammograms with PIFU. In addition, two breast cancer studies reported how recurrences were detected and found a mixture of referral mechanisms including via GPs, specialist nurses, after clinic visit and emergency admission.^23^, ^26^ This limited evidence suggests that detection of recurrence is not largely dependent on routine clinic visits.

One RCT in colorectal cancer found no difference in recurrence and survival when foregoing scheduled visits^28^; another study found fewer outpatient visits with PIFU, with no overall differences in non‐routine clinic visits, clinic visits initiated by patients, outpatient nurse visits and telephone consultations.^8^ Some patient involvement and satisfaction scores were higher with PIFU in the latter study. One RCT in endometrial cancer found significantly less fear of cancer with routine FU (though no difference in clinical level severity of FCR), and significantly fewer hospital examinations in the PIFU arm.^30^ There were no significant differences in cancer‐related visits to the GP or to private gynaecologists. The RCT in melanoma found more diagnoses of recurrence or new melanoma in the patient‐led surveillance group; this difference was statistically significant when only unscheduled (patient initiated) visits were considered.^31^ There were increased levels of confidence around skin self‐examination, but no differences in psychological morbidity. In this study patient‐led surveillance was an add‐on to usual care in one trial arm, and the number of clinic visits was increased in the PIFU + routine FU arm.

FU models are complex and comprised of different components, consequently ‘PIFU’ or ‘routine FU’ were not always comparable between studies. The melanoma study was the only study to include routine FU in both arms, with patient‐led surveillance added in to one arm, so explored the effect of adding an element of PIFU but without a reduction in routine visits,^31^ while two of the breast cancer studies compared PIFU with routine FU + PIFU.^24^, ^25^ While PIFU mostly entails no, or fewer, scheduled visits and instead provides on‐demand access to clinics, there may be other components included either in PIFU and/or in routine FU. Examples include ePROS collected in the PIFU arm of a breast cancer study (which in turn could trigger contacts)^27^ or a psycho‐educational self‐management programme in both PIFU and routine FU arms, also in a breast cancer study.^25^ A study on ovarian cancer suggested that virtual visits which included patient reported symptoms alongside tumour marker testing and imaging may be a suitable approach as recurrence was not primarily detected through in‐person physical examination.^32^ Other PIFU components identified in the included studies were education on signs and symptoms of recurrence, instructions on how to self‐refer, advice to self‐examine monthly (e.g. for breast cancer), availability of specialised nurses for phone calls or meetings and ability of nurses to instigate investigations.

Inclusion (and frequency) of regular imaging or testing in a FU programme is specific to type of cancer and underlying risk of recurrence. All breast cancer studies in this review included scheduled mammograms in both arms and the colorectal studies included either scheduled CT scans in both arms,^8^ or instructions to leave regular faecal samples with a nurse in the PIFU arm.^28^ There is evidence to suggest that there is a survival benefit from asymptomatic recurrence detection based on imaging or other diagnostic tests, for example in gastric cancer,^33^ breast cancer,^34^ bladder cancer^35^ or colon cancer.^36^

Foregoing routine clinic visits as part of PIFU does not necessarily translate to a reduction in cost, as PIFU may entail greater use of, for example, specialist nurses, and there remains uncertainty around whether patient‐initiated appointment systems specifically lead to reduced service utilisation or costs.^3^ A systematic review of cost‐effectiveness studies of cancer FU found that intensive, hospital‐based FU is unlikely to be beneficial in breast cancer, but that intensive FU which could be conducted outside of a hospital setting is likely to be cost‐effective in colorectal cancer.^37^ However, this review did not look at PIFU specifically. Clinician perception may play a role in how PIFU is implemented, and resistance to change could be a barrier to implementation of PIFU, whether in a trial context or in a real‐life setting.^11^ In one colorectal cancer study, 38% in the PIFU arm had planned routine clinic visits in non‐compliance with the protocol and/or because it was deemed clinically necessary.^8^ There is a concern with PIFU that patients may not always request urgent appointments despite recognising symptoms,^38^, ^39^ and in such cases regular follow‐up may aid access to specialists.^16^, ^40^

There are limitations to this review. Identification of RCTs was based primarily on a search for existing systematic reviews and checking of reference lists rather than a search for primary studies. The large number of systematic reviews identified, and the overlap between these in terms of included RCTs as well as an updated search for more recent RCTs make it unlikely that any RCTs were missed.

All included studies had some risk of bias concerns, particularly with regard to patient reported outcomes. Studies were also underpowered and had too short a FU for long‐term outcomes such as recurrence and mortality. Populations in the included studies were unlikely to have been representative of a wider cancer population. Recruitment rates, where reported, ranged from 31% to 93% of those eligible. Several studies reported differences between participants and non‐participants, which suggests that PIFU may be less accessible or acceptable to some patient groups. These might be patients who are older, more severely ill or who are not comfortable with using digital technologies where these play a part in PIFU. We know that RCTs often have stringent inclusion criteria which do not reflect the heterogeneity of real‐world populations.^41^ In the trials included in this review, patients were deemed to be ineligible where they had, for example, psychosocial problems,^23^, ^27^ were not fluent in the native language of where the study was set,^8^, ^24^ had stage III or IV breast cancer,^25^ had cognitive impairment,^8^, ^27^, ^31^ did not have computer skills or a compatible smartphone,^27^, ^31^ had a tumour with high risk histology or were undergoing treatment with radiation therapy,^30^ had a synchronous cancer^8^ or inflammatory bowel disease.^28^

Studies were set in the United Kingdom (*n* = 4), Denmark (*n* = 3), Sweden (*n* = 2) and Australia (*n* = 1). There is currently substantial interest in PIFU in the United Kingdom where one of the National Health Service's (NHS) priorities is supporting providers to implement PIFU for a range of conditions. NHS guidance suggests that PIFU is suitable for oncology, with the proviso that PIFU may not be appropriate for all patients, for example, those with complex health issues, those unable to use the service easily or where there are clinical requirements for a patient to be seen regularly.^42^ The guidance also notes that blended follow‐up comprised of PIFU and planned follow‐ups may be suitable for cancer pathways.^42^ PIFU is not seen by health professionals as suitable for all types of patients or all cancers, and feasibility might depend on risk of recurrence and ability of patients to recognise recurrence.^4^, ^11^ It is currently not known how PIFU might affect health inequalities, as participation in studies has mostly included those at lower risk and better able to initiate contact, and there is a concern that for some cancers those patients less likely to engage may also be those at higher risk of recurrence.^11^, ^43^

A recent systematic review of acceptability of PIFU undertaken by the authors of this review found that PIFU is mostly seen as acceptable by women treated for breast or endometrial cancer, but may not be acceptable to a smaller proportion of patients.^44^ Facilitators for PIFU included convenience, patients gaining control over their own health and avoidance of anxiety inducing routine appointments; barriers included a loss of reassurance (from routine appointments), difficulties accessing PIFU (especially by non‐English language speakers) and avoidance or fear of self‐examination. Participants in the included studies were unlikely to be representative of a general cancer population and the review concluded that more representative evidence from a wider range of cancers was needed.

Several ongoing trials are exploring versions of PIFU compared with routine FU. The RCT in rectal cancer included above is still ongoing,^8^ and a larger melanoma RCT is planned based on the pilot trial included above.^31^ Other ongoing or planned studies include the PETNECK2,^45^ INFLUENCE^46^ and DeintensiF^47^ trials in head and neck cancer, and the DISTANCE trial^48^ in colorectal cancer. Results from these and other studies will help to address some of the current evidence gaps.

In conclusion, there is limited evidence from breast cancer studies that type of follow‐up may not affect detection of recurrence, mortality, quality of life, psychological morbidity or patient dissatisfaction, and that PIFU may reduce the number of some clinic visits thus potentially increasing efficiency of care. While there is insufficient evidence on PIFU for other types of cancers, the evidence suggests a potential role for PIFU in melanoma for recurrence detection.

Half the included studies are over 10 years old and may not adequately reflect current practice. Future studies will need to be larger and with longer FU to determine any effect on recurrence and mortality and should also consider representativeness of included patients. A further consideration will be whether to include regular imaging or other monitoring into either FU arm, which will likely be informed by type of cancer and underlying risk of recurrence. Finally, future studies might want to consider how PIFU can be integrated into existing or planned self‐management or survivorship care programmes.

## AUTHOR CONTRIBUTIONS

**Janine Dretzke:** Formal analysis (lead); investigation (equal); methodology (equal); project administration (lead); validation (equal); writing – original draft (lead); writing – review and editing (equal). **Talhah Chaudri:** Investigation (supporting); writing – review and editing (equal). **Rishab Balaji:** Investigation (supporting); writing – review and editing (equal). **Hisham Mehanna:** Conceptualization (equal); funding acquisition (lead); methodology (equal); supervision (equal); writing – review and editing (equal). **Paul Nankivell:** Conceptualization (equal); funding acquisition (equal); methodology (equal); supervision (equal); writing – review and editing (equal). **David J. Moore:** Formal analysis (equal); methodology (equal); validation (equal); writing – original draft (equal); writing – review and editing (equal).

## FUNDING INFORMATION

This work was funded by a National Institute for Health and Care Research (NIHR) Programme Grant for Applied Research (NIHR200861).

## CONFLICT OF INTEREST STATEMENT

None of the authors report any conflicts of interest.

## Supporting information


Data S1

## Data Availability

Extracted data from published articles available in Supplementary material. All published articles are in the public domain.
