# Supplementary material for: A systematic review of the effectiveness of patient‐initiated follow‐up after cancer
Source: Cancer Med. 2023 Aug 21;12(18):19057–71. doi: 10.1002/cam4.6462 (PMC10557867; doi:10.1002/cam4.6462)
Supplement: Supplementary file 1 — Data S1 [file CAM4-12-19057-s001.zip › cam46462-sup-0001-Supinfo/Suppl 1 PETNECK2 Research Team (revised).docx]

**Additional members of the PETNECK2 Research Team - in alphabetical order by first name**

Ahmad Abou-Foul (Institute of Cancer and Genomic Sciences, University of Birmingham) A.Abou-Foul@bham.ac.uk

Andreas Karwath (Institute of Cancer and Genomic Sciences, University of Birmingham) A.Karwath@bham.ac.uk

Ava Lorenc (Bristol Medical School, Bristol Population Health Science Institute) ava.lorenc@bristol.ac.uk

Barry Main (University Hospitals Bristol and Weston NHS Trust) B.G.Main@bristol.ac.uk

Claire Gaunt (Cancer Clinical Trials Unit, University of Birmingham) C.H.Gaunt@bham.ac.uk

Colin Greaves (School of Sport, Exercise and Rehabilitation Sciences, University of Birmingham) C.J.Greaves@bham.ac.uk

Eila Watson (Oxford School of Nursing and Midwifery, Oxford Brookes University) ewatson@brookes.ac.uk

Evaggelia Liaskou (Cancer Clinical Trials Unit, University of Birmingham) E.Liaskou@bham.ac.uk

Georgios Gkoutos (Institute of Cancer and Genomic Sciences, University of Birmingham) G.Gkoutos@bham.ac.uk

Gozde Ozakinci (Health Psychology Research Group, University of Stirling) Go7@stir.ac.uk

Jane Wolstenholme (Nuffield Department of Population Health, University of Oxford) jane.wolstenholme@dph.ox.ac.uk

Jo Brett (Department of Midwifery, Community and Public Health, Oxford Brookes University) jbrett@brookes.ac.uk

Joan Duda (School of Sport, Exercise and Rehabilitation Sciences, University of Birmingham) J.L.DUDA@bham.ac.uk

Lauren Matheson (Oxford Institute of Nursing, Midwifery and Allied Health Research, Oxford Brookes University) l.matheson@brookes.ac.uk

Louise-Rae Cherrill (Cancer Clinical Trials Unit, University of Birmingham) l.cherrill@bham.ac.uk

Marcus Jepson (Bristol Medical School, Bristol Population Health Science Institute) Marcus.Jepson@bristol.ac.uk

Mary Wells (Imperial College Healthcare NHS Trust) Mary.Wells5@nhs.net

Melanie Calvert (Institute of Applied Health Research, University of Birmingham) M.Calvert@bham.ac.uk

Philip Kiely (University Hospitals Bristol and Weston NHS Foundation Trust) Philip.Kiely@UHBristol.nhs.uk

Piers Gaunt (Cancer Clinical Trials Unit, University of Birmingham) P.Gaunt@bham.ac.uk

Saisakul Chernbumroong (Institute of Cancer and Genomic Sciences, University of Birmingham) S.Chernbumroong@bham.ac.uk

Saloni Mittal (Institute of Head and Neck Studies and Education, Institute of Cancer and Genomic Sciences, University of Birmingham) s.mittal.2@bham.ac.uk

Steve Thomas (Bristol Dental School, University of Bristol) Steve.Thomas@bristol.ac.uk

Stuart Winter (Nuffield Department of Surgical Sciences, University of Oxford) Stuart.Winter@ouh.nhs.uk

Wailup Wong (East and North Hertfordshire NHS Trust, Mount Vernon Cancer Centre) wailup.wong@nhs.net
