# Supplementary material for: A systematic review of the effectiveness of patient‐initiated follow‐up after cancer
Source: Cancer Med. 2023 Aug 21;12(18):19057–71. doi: 10.1002/cam4.6462 (PMC10557867; doi:10.1002/cam4.6462)
Supplement: Supplementary file 1 — Data S1 [file CAM4-12-19057-s001.zip › cam46462-sup-0001-Supinfo/Suppl 2 Sample search strategy.docx]

**Sample search strategy Embase**

1 (patient$ adj2 initiat$).ti,ab.

2 (patient$ adj2 prefer$).ti,ab.

3 (patient$ adj2 request$).ti,ab.

4 (patient$ adj2 prompt$).ti,ab.

5 (patient$ adj2 trigger$).ti,ab.

6 (patient$ adj2 led).ti,ab.

7 (patient$ adj2 driv$).ti,ab.

8 (patient$ adj2 generat$).ti,ab.

9 (survivor$ adj2 initiat$).ti,ab.

10 (survivor$ adj2 prefer$).ti,ab.

11 (survivor$ adj2 request$).ti,ab.

12 (survivor$ adj2 prompt$).ti,ab.

13 (survivor$ adj2 trigger$).ti,ab.

14 (survivor$ adj2 led).ti,ab.

15 (survivor$ adj2 driv$).ti,ab.

16 (survivor$ adj2 generat$).ti,ab.

17 self-refer$.ti,ab.

18 help seek$.ti,ab.

19 helpseek$.ti,ab.

20 follow up$.ti,ab.

21 clinic$.ti,ab.

22 consultation$.ti,ab.

23 appointment$.ti,ab.

24 monitor$.ti,ab.

25 aftercare.ti,ab.

26 refer$.ti,ab.

27 examination$.ti,ab.

28 surveillance.ti,ab.

29 watchful waiting.ti,ab.

30 survivorship care.ti,ab.

31 survivorship plan$.ti,ab.

32 (follow-up adj2 strateg$).ti,ab.

33 (follow-up adj2 model$).ti,ab.

34 (follow-up adj2 system$).ti,ab.

35 (follow-up adj2 protocol$).ti,ab.

36 (follow-up adj2 pathway$).ti,ab.

37 (individuali#ed adj2 follow-up).ti,ab.

38 (targeted adj2 follow-up).ti,ab.

39 personali#ed follow-up.ti,ab.

40 (follow-up adj2 prefer$).ti,ab.

41 personali#ed care.ti,ab.

42 individuali#ed care.ti,ab.

43 personali#ed survivorship.ti,ab.

44 individuali#ed survivorship.ti,ab.

45 (prefer$ adj2 survivorship).ti,ab.

46 cancer$.ti,ab.

47 neoplasm$.ti,ab.

48 carcinoma$.ti,ab.

49 metastas$.ti,ab.

50 malignan$.ti,ab.

51 tumo?r$.ti,ab.

52 exp malignant neoplasm/

53 1 or 2 or 3 or 4 or 5 or 6 or 7 or 8 or 9 or 10 or 11 or 12 or 13 or 14 or 15 or 16 or 17 or 18 or 19

54 20 or 21 or 22 or 23 or 24 or 25 or 26 or 27 or 28 or 29 or 30 or 31

55 53 and 54

56 32 or 33 or 34 or 35 or 36 or 37 or 38 or 39 or 40 or 41 or 42 or 43 or 44 or 45

57 55 or 56

58 46 or 47 or 48 or 49 or 50 or 51 or 52

59 57 and 58

60 limit 59 to (meta analysis or "systematic review")

61 limit 59 to "reviews (maximizes specificity)"

62 60 or 61

63 (systematic review or meta-analysis).ti.

64 59 and 63

65 62 or 64
