# Supplementary material for: A systematic review of the effectiveness of patient‐initiated follow‐up after cancer
Source: Cancer Med. 2023 Aug 21;12(18):19057–71. doi: 10.1002/cam4.6462 (PMC10557867; doi:10.1002/cam4.6462)
Supplement: Supplementary file 1 — Data S1 [file CAM4-12-19057-s001.zip › cam46462-sup-0001-Supinfo/Suppl 3 Risk of bias assessment (revised).docx]

**Risk of bias assessment RCTs (Cochrane Risk of Bias Tool - 2)**

| **Signalling question** | **Brown 2002^23^** | **Gulliford 1997^24^** | **Koinberg 2004^7^** | **Sheppard 2009^26^** | **Kirshbaum 2017^25^** | **Riis 2020^27^** | **Jeppesen 2018^30^** | **Ohlsson 1995^28^** | **Hovdenak Jacobsen 2021^8,29^** | **Ackermann 2022^31^** |
| --- | --- | --- | --- | --- | --- | --- | --- | --- | --- | --- |
| *Outcome assessment undertaken for* | EORTEC QLQ-30 | Patient preferences for FU | HADS | General Health Questionnaire (GHQ12) | EORTEC QLQ-30 | Patient Experience Questionnaire | Fear of cancer recurrence inventory | Tumour recurrence | Patient satisfaction | Fear of recurrence |
| *1.1 Was the allocation sequence random?* | Y, PY  *“Random number list generated by*  *the Medical Statistics Department at Southampton*  *General Hospital.”* | NI | Y, Y  *“Randomisation was achieved by means of telephone contact with an external secretariat. The*  *random selection was computer-generated and*  *stratified by centre. The block size was unknown*  *to the study co-ordinators at the centres.”* | Y, Y  *“Sequential series of sealed envelopes containing computer generated random assignments*  *produced externally”* | NI | NI | Y, Y  *“…computer-based system stratified according to healthcare centre. Randomisation was performed centrally with a block size of ten within each hospital.”* | NI | PY  *“…participants were 1:1 block randomised in blocks of*  *50 patients, stratified by centre, sex and treatment type.”* | Y, Y  *“participants were randomized 1:1 to either the intervention or the control group using*  *an interactive voice recognition system provided by an off-site telephone randomization service”* |
| *1.2 Was the allocation sequence concealed until participants were enrolled and assigned to interventions?* |  | NI |  |  | NI | NI |  | NI | NI |  |
| *1.3 Did baseline differences between intervention groups suggest a problem with the randomization process?* | PN  Not significantly  different in terms of age  or time since surgery. | PN  Similar age distribution, duration since diagnosis and primary disease stage. | PN  Similar characteristics across groups. | PN  *“The two groups were well matched in terms of a number of*  *demographic characteristics that might be potential prognostic*  *variables.”* | NI  Demographic, social and co-morbidity factors not recorded (other than age). | PN  Participants did not differ by clinical or sociodemographic  Characteristics. | PN  Mostly similar patient characteristics across study arms. Slightly more patients with higher education in PFU arm (33% vs 21%). | PN  Reasonably similar characteristics across groups. | PN  Reasonably similar characteristics across groups. | PN  *“Clinical and demographic characteristics were generally*  *well balanced across the randomized intervention and control group.”* |
| *Risk-of-bias judgement Domain 1* | LOW RISK | SOME CONCERNS | LOW RISK | LOW RISK | SOME CONCERNS | SOME CONCERNS | LOW RISK | SOME CONCERNS | SOME CONCERNS | LOW RISK |
| *2.1. Were participants aware of their assigned intervention during the trial?* | Y, Y  Blinding not possible. | Y, Y  Blinding not possible. | Y, Y  Blinding not possible. | Y, Y  Blinding not possible. | Y, Y  Blinding not possible. | Y, Y  Blinding not possible. | Y, Y  Blinding not possible. | Y, Y  Blinding not possible. | Y, Y  Blinding not possible. | Y, Y  Blinding not possible. |
| *2.2. Were carers and people delivering the interventions aware of participants' assigned intervention during the trial?* |  |  |  |  |  |  |  |  |  |  |
| *2.3. If Y/PY/NI to 2.1 or 2.2: Were there deviations from the intended intervention that arose because of the trial context?* | PN  No evidence to suggest that delivery of interventions being influenced by trial context. | PN  No evidence to suggest that delivery of interventions being influenced by trial context. | PN  No evidence to suggest that delivery of interventions being influenced by trial context.  *“The interventions remained identical over*  *time, and there was no patient crossover.”* | PN  No evidence to suggest that delivery of interventions being influenced by trial context. | PN  No evidence to suggest that delivery of interventions being influenced by trial context. | PN  No evidence to suggest that delivery of interventions being influenced by trial context. | PN  No evidence to suggest that delivery of interventions being influenced by trial context.  *“Intervention evaluated in real-life-setting”.* | PN  No evidence to suggest that delivery of interventions being influenced by trial context. | PY  38% in PIFU arm had planned follow-up visits, some due to non-compliance of the hospital with the protocol. 15% in routine arm did not receive a routine visit. | PN  No evidence to suggest that delivery of interventions being influenced by trial context. |
| *2.4 If Y/PY to 2.3: Were these deviations likely to have affected the outcome?* | N/A | N/A | N/A | N/A | N/A | N/A | N/A | N/A | PY  Would affect number of contacts, which in turn may affect other outcomes (e.g. satisfaction) | N/A |
| *2.5. If Y/PY/NI to 2.4: Were these deviations from intended intervention balanced between groups?* | N/A | N/A | N/A | N/A | N/A | N/A | N/A | N/A | N  More crossover from PIFU to routine follow-up arm. | N/A |
| *2.6 Was an appropriate analysis used to estimate the effect of assignment to intervention?* | NI  No details given on how missing data was handled. | NI  No details given on how missing data was handled. | Y  Intention-to-treat analysis. | PN  Data from completers only included in analysis (not ITT) | NI  Unclear if intention-to-treat analysis used. Data imputation for some but not all missing values. | N  Per protocol analysis. | Y  “For all analyses, a modified intention-to-treat approach was applied, as only women who completed the first and fourth questionnaires were included in the analyses.”  “Questionnaires were discarded if (1) more than half of the data were missing, (2) two or more subscales were completely missing, or (3) a ‘0’-response pattern for the entire FCRI was used. Otherwise, missing data were imputed by the mean subscale score on a person level.” | NI  No details on loss to FU/analysis strategy. | PN  Appears analysis based on available data only. | N  Stated that ITT but appears only for primary outcomes.  *61% (intervention group) and 71% (control group)*  *completed the 6-month questionnaire and were included in the analyses of the patient-reported secondary outcomes.* |
| *2.7 If N/PN/NI to 2.6: Was there potential for a substantial impact (on the result) of the failure to analyse participants in the group to which they were randomized?* | Unclear/NI  1/31 patient (3%) crossed over from the routine clinic to GP FU. 8% not contributing to data at later time points. | NI  3/196 (1.5%) patients crossed over to an alternative FU regime (unclear from which group). Unclear how many patients contributing to preferences. | N/A | Unclear/NI  Appeared that no patients in PIFU group crossed over to routine care during study period. 10% not contributing to data. | Unclear/NI | Unclear/NI | N/A | PN  3/54 (6%) asymptomatic patients (PIFU group) returned once for a control examination (but did not fully cross over to routine FU arm). Appears no loss to FU for this outcome. | NI | Unclear/NI  Info given on reasons for withdrawals but no info on responders vs non-responders. |
| *Risk-of-bias judgement Domain 2** | HIGH RISK | HIGH RISK | LOW RISK | HIGH RISK | HIGH RISK | HIGH RISK | LOW RISK | SOME CONCERNS | HIGH RISK | HIGH RISK |
| *3.1 Were data for this outcome available for all, or nearly all, participants randomized?* | N  PIFU: 28/30 (93%)  Control: 28/31 (90%) | NI | N  At 60 months: 30% and 28% of patients not observed (anxiety and depression sub-scales). | N  Loss to FU/non-completers: 10% (23/237) | N  27% lost to FU by 2 years. | N  7.5% not included in analysis. | N  PIFU: 79/105 analysed (75%)  Control: 77/107 analysed (72%) | Y  Recurrence data available for all. | N (up to 32% of missing responses depending on questionnaire item) | N  *31% missing in control group, 39% missing intervention group* |
| *3.2 If N/PN/NI to 3.1: Is there evidence that the result was not biased by missing outcome data?* | N  No evidence that analysis methods correct for bias; no sensitivity analyses. | N  No evidence that analysis methods correct for bias; no sensitivity analyses. | N | N  No evidence that analysis methods correct for bias; no sensitivity analyses. | N  No evidence that analysis methods correct for bias; no sensitivity analyses. | N  No evidence that analysis methods correct for bias; no sensitivity analyses. | N  No evidence that analysis methods correct for bias; no sensitivity analyses. | N/A | N  No evidence that analysis methods correct for bias; no sensitivity analyses. | N  No evidence that analysis methods correct for bias; no sensitivity analyses. |
| *3.3 If N/PN to 3.2: Could missingness in the outcome depend on its true value?* | NI | NI | NI | NI | NI | NI | NI | N/A | NI | NI |
| *3.4 If Y/PY/NI to 3.3: Is it likely that missingness in the outcome depended on its true value?* | NI | NI | NI | NI | NI | NI | NI | N/A | NI | NI |
| *Risk-of-bias judgement Domain 3* | HIGH RISK | HIGH RISK | HIGH RISK | HIGH RISK | HIGH RISK | HIGH RISK | HIGH RISK | LOW RISK | HIGH RISK | HIGH RISK |
| *4.1 Was the method of measuring the outcome inappropriate?* | N  *“The EORTC QLQ-C30 is…proven to be reliable and*  *Valid.”* | PN  Unclear how additional questions on MRC QoL questionnaire were derived. | N  *“HAD has been found to be a reliable*  *instrument in the setting of a hospital outpatient*  *clinic.”* | N  GHQ-12 widely validated. | N  EORTC-QLQ widely validated. | N  PEQ widely validated. | N  *“The English version of the FCRI has*  *demonstrated sound psychometric properties, including high*  *internal consistency (Cronbach a 0.71–0.94), test–retest reliability*  *(r 0.56–0.87), and construct validity.”* | N  Detailed definition given of how recurrences defined/detected. | PN  Items on patient satisfaction selected from validated questionnaire. | PN  Melanoma specific version of the Fear of Cancer Recurrence  Inventory Severity subscale |
| *4.2 Could measurement or ascertainment of the outcome have differed between intervention groups?* | PN  Data collection at same time-points and seen by research nurse if possible. | PN  Data collection at same time-points. | PN  Same methods and time-points of data collection. | PN  Same methods and time-points of data collection. | PN  Data collection at same time-points. | PN  Same methods and time-points of data collection. | PN  Data collection at same time-points. | PN  Not stated but unlikely to differ between patients. | PN  Not stated but unlikely to differ between patients. | PN  Not stated but unlikely to differ between patients. |
| *4.3 If N/PN/NI to 4.1 and 4.2: Were outcome assessors aware of the intervention received by study participants?* | Y  Patient-reported outcome. | Y  Patient-reported outcome. | Y  Patient-reported outcome. | Y  Patient-reported outcome. | Y  Patient-reported outcome. | Y  Patient-reported outcome. | Y  Patient-reported outcome. | NI  Unclear if outcome assessors know which FU regime patients were on. | Y  Patient-reported outcome. | Y  Patient-reported outcome. |
| *4.4 If Y/PY/NI to 4.3: Could assessment of the outcome have been influenced by knowledge of intervention received?* | Y  Patients aware of treatment allocation. | Y  Patients aware of treatment allocation. | Y  Patients aware of treatment allocation. | Y  Patients aware of treatment allocation. | Y  Patients aware of treatment allocation. | Y  Patients aware of treatment allocation. | Y  Patients aware of treatment allocation. | PN  Objective outcome. | Y  Patients aware of treatment allocation. | Y  Patients aware of treatment allocation. |
| *4.5 If Y/PY/NI to 4.4:* *Is it likely that assessment of the outcome was influenced by knowledge of intervention received?* | NI | NI | NI | NI | NI | NI | NI | N/A | NI | NI |
| *Risk-of-bias judgement*  *Domain 4* | HIGH RISK | HIGH RISK | HIGH RISK | HIGH RISK | HIGH RISK | HIGH RISK | HIGH RISK | LOW RISK | HIGH RISK | HIGH RISK |
| *5.1 Were the data that produced this result analysed in accordance with a pre-specified analysis plan that was finalized before unblinded outcome data were available for analysis?* | NI  No trial protocol identified. | NI  No trial protocol identified. | NI  No trial protocol identified. | NI  *“The study was originally commenced in 2005 prior to the*  *requirement for trial registration.”* | NI  No trial protocol identified. | PY  Trial protocol available. | PY  Trial protocol available. Study assessed is an interim report of some outcomes at 10 months. | NI  No trial protocol identified. | PY  Trial protocol available. Study assessed is an interim report of some outcomes at 12 months. | PY  Trial protocol available. |
| *Is the numerical result being assessed likely to have been selected, on the basis of the results, from...* | | | | | | | | |  |  |
| *5.2. ... multiple eligible outcome measurements (e.g. scales, definitions, time points) within the outcome domain?* | PN  Appears all results reported. | PN  NB-interim report of only some outcomes. | PN  Appears all results reported | PN  Appears all results reported | PN  Appears all results reported | PN  Appears all results reported | PN  All results reported at all time-points (mean and % with clinical level). | PN  Appears all results reported. | PN  NB-interim report of only some outcomes | PN  Appears all results reported. |
| *5.3 ... multiple eligible analyses of the data?* | PN  No evidence of results selection. | PN  No evidence of results selection. | PN  No evidence of results selection. | PN  No evidence of results selection. | PN  No evidence of results selection. | PN  No evidence of results selection. | PN  No evidence of deviation from protocol. | PN  No evidence of results selection. | PN  No evidence of results selection. | PN  No evidence of results selection. |
| *Risk-of-bias judgement*  *Domain 5* | SOME CONCERNS | SOME CONCERNS | SOME CONCERNS | SOME CONCERNS | SOME CONCERNS | LOW RISK | LOW RISK | SOME CONCERNS | LOW RISK | LOW RISK |
| *OVERALL RISK OF BIAS* | HIGH RISK | HIGH RISK | HIGH RISK | HIGH RISK | HIGH RISK | HIGH RISK | HIGH RISK | SOME CONCERNS | HIGH RISK | HIGH RISK |

*For domain 2 (Risk of bias due to deviations from the intended interventions), the effect of assignment to intervention (intention- to-treat analysis) was assessed.
