# Supplementary material for: A systematic review of the effectiveness of patient‐initiated follow‐up after cancer
Source: Cancer Med. 2023 Aug 21;12(18):19057–71. doi: 10.1002/cam4.6462 (PMC10557867; doi:10.1002/cam4.6462)
Supplement: Supplementary file 1 — Data S1 [file CAM4-12-19057-s001.zip › cam46462-sup-0001-Supinfo/Suppl 4 Results tables (revised).docx]

**Selected results**

***Breast cancer***

**Recurrence (and how detected) and mortality**

| **Study** | **PIFU** | **Control** | **Summary measure, p-value** | **Direction of effect** |
| --- | --- | --- | --- | --- |
| Brown 2002, UK^23^  n=61  PIFU n=30  Control n=31 | 2 recurrences (6.7%)  Both referred by GP | 2 recurrences (6.5%)  One diagnosed at outpatient clinic and one referred by GP (after contacting breast care nurse). | No summary measure or p-values reported. | No difference in number of recurrences (low numbers). |
| Koinberg 2004, Sweden^7^  n=264  PIFU n=133  Control n=131 | Number of:  Loco regional recurrence:12 (9%)  Distant metastasis:9 (6.8%)  First recurrence (local or distant):17 (12.8%)  All cause death:14 (10.5%) | Number of:  Loco regional recurrence:8 (6.1%)  Distant metastasis:9 (6.9%)  First recurrence (local or distant):16 (12.2%)  All cause death:14 (10.7%) | Difference in Kaplan-Meier estimates (routine FU-PIFU, 95% CI)  Time to locoregional recurrence: 3% (-2,8) 3-yr estimate (p-value not stated)  Time to distant metastasis:  0.6% (-6, 5) 3-yr estimate (p-value not stated)  Time to first recurrence:  2% (-5,9) 3yr estimate (p-value not stated)  Time to all cause death:  -0.3% (-10,9) 5yr estimate (p=0.6) | No statistically significant differences between the groups.  Insufficient number of events for 5-year estimates of being free from loco-regional recurrence or distant metastases. |
| Sheppard 2009, UK^26^  n=237  PIFU n=107*  Control n=107*  *only number analysed by group | 5 (4%) recurrences (1 emergency admission, 1 after mammography, 1 GP emergency admission, 2 after contact with BCN and bone scan) | 4 recurrences (4%) (2 emergency admission, 1 patient identified (but waited until next review), 1 GP emergency admission | No summary measure or p-values reported. | Similar number of recurrences (low numbers).  No further recurrences detected on clinical review at end of study of all patients in PIFU arm. |

**EORTEC QLQ-C30***

| **Study** | **PIFU** | **Control** | **Summary measure, p-value** | **Direction of effect** |
| --- | --- | --- | --- | --- |
| Brown 2002, UK^23^  n=61  PIFU n=30  Control n=31 | Median (IQR) (raw scores)  No summary scores  **Baseline, 12 months**  Physical: 5 (1), 5 (1)  Role :2(0), 2(0)  Cognitive: 2(1), 3(1)  Emotional: 5(3), 5(3)  Social: 2(0), 2(0)  Global QoL:12(3.75), 11(2)  Fatigue: 4(2), 4(1.5)  Nausea/vomiting: 2(0), 2(0)  Pain:1 (0.25), 1(1)  Dyspnoea:1(0), 1(1)  Sleep disturbance: 1.5(1), 1.5(1)  Appetite loss:1(0), 1(0)  Constipation: 1(0), 1(0)  Diarrhoea: 1(0), 1(0)  Financial impact: 1(0), 1(0) | Median (IQR) (raw scores)  No summary scores  **Baseline, 12 months**  Physical: 5(1), 6(2)  Role: 2(0), 2(0)  Cognitive: 3(1), 3(1)  Emotional:6(4), 6(3.5)  Social: 2(1.25), 2(0.25)  Global QoL:11(4.5), 10(3)  Fatigue: 4(3), 5(2)  Nausea/vomiting: 2(0), 2(0)  Pain: 1 (1), 1(1)  Dyspnoea: 1 (1), 1(1)  Sleep disturbance: 1(1), 2(1)  Appetite loss:1(0), 1(0)  Constipation: 1(0), 1(0)  Diarrhoea: 1(0), 1(0)  Financial impact: 1(0), 1(0) | No summary measure reported, p=0.023 (sub-scale social functioning) | No significant differences between groups at each time point, except for social functioning sub-scale: same median scores, but routine FU scores more varied and higher maximum score.  Time of data collection had no influence on subscale scores.  *NB 6 month results also presented, not extracted.* |
| Kirshbaum 2017, Australia & UK^25^  n=112  PIFU n=56  Control n=56 | Mean (SD) of transformed score+  Baseline, 24 months  Physical: 86.5 (17.5), 86.7 (19.3)  Role: 75.6 (25.8), 82.2 (22.7)  Cognitive: 78.5 (20.3), 76.9 (21.4)  Emotional: 75.8 (17.9), 75.3 (24.8)  Social: 79.6 (23.8), 86.6 (20.8)  Global QoL: 70.7 (18.1), 69.9 (20.5)  Fatigue: 33.0 (17.2), 27.2 (24.6)  Nausea/vomiting: 4.4 (8.9), 7.8 (16.2)  Pain:22.7 (21.6), 15.7 (20.8)  Dyspnoea:12.6 (20.7), 14.9 (26.1)  Sleep disturbance: 34.5 (31.5), 32.2 (28.8)  Appetite loss:6.7 (16.1), 5.6 (19.7)  Constipation: 17.2 (26.2), 18.4 (30.3)  Diarrhoea: 6.7 (16.1), 6.7 (16.1)  Financial impact: 13.8 (26.0), 5.7 (21.9) | Mean (SD) of transformed score+  Baseline, 24 months  Physical: 86.1 (14.0), 86.1 (15.3)  Role: 78.2 (27.6), 81.5 (28.7)  Cognitive: 79.5 (24.9), 80.3 (24.4)  Emotional: 77.9 (17.7), 75.4 (27.4)  Social: 82.9 (22.5), 85.5 (21.7)  Global QoL:72.9 (18.8), 75.0 (19.8)  Fatigue: 30.9 (26.9), 30.2 (26.9)  Nausea/vomiting: 3.8 (8.1), 5.6 (12.9)  Pain:18.8 (28.1), 17.9 (27.4)  Dyspnoea:16.7 (27.0), 14.8 (24.5)  Sleep disturbance: 34.3 (33.3), 35.2 (32.8)  Appetite loss:9.9 (17.3), 7.2 (13.9)  Constipation: 14.5 (26.3), 12.8 (26.1)  Diarrhoea: 7.2 (16.0), 10.8 (19.3)  Financial impact:10.3 (25.5), 10.3 (27.7) | No summary measure or p-values reported. | Type of FU not found to be a statistically significant predictor on multilevel linear regression modelling.  *NB 6, 12 and 18 month results also presented (not extracted)* |
| Riis 2020, Denmark^27^  n=129  PIFU n=62  Control n=67 | Summary score  Mean (95% CI), estimated from graph  Baseline: 85.0 (81.6, 87.9)  3 months: 85.7 (82.5, 88.8)  6 months: 86.5 (84.1, 89.5)  9 months:87.6 (84.9, 90.9)  12 months: 88.4 (85.4, 91.2)  15 months: 89.0 (86.8, 91.6)  18 months:88.2 (85.7, 91.5)  21 months: 89.3 (86.5, 92.0)  24 months:89.0 (86.5, 91.7) | Summary score  Mean (95% CI), estimated from graph  Baseline: 82.4 (78.9, 85.4)  3 months: 86.0 (83.2, 88.5)  6 months:86.8 (84.1, 89.3)  9 months:86.0 (83.2, 88.2)  12 months: 87.9 (85.2, 89.8)  15 months:87.9 (85.4, 89.5)  18 months:88.2 (85.7, 90.1)  21 months:89.0 (86.5, 90.9)  24 months:87.9 (85.7, 90.1) | No summary measure or p-values reported. | No significant differences between groups for the summary scores or subscales (or symptoms) measured.  Longitudinal linear mixed effects models did not show any significant difference  between the two groups. |

*** *European Organisation for Research and Treatment of Cancer (**EORTC) QLQ-C30; 30 item questionnaire; includes five functional scales (physical, emotional, cognitive, social, and role), three symptom scales (fatigue, pain, and nausea/vomiting), a global health related quality of life scale, and six single items on common symptoms.* *Twenty-seven out of the 30 items can be combined into a summary score estimating health related quality of life based on all five functioning scales and all reported symptoms.* *For the functional and the global QoL scales, a higher score indicates better health. For the symptom scales, a higher score indicates greater symptom burden.*

**EORTEC QLQ-BR23***

| **Study** | **PIFU** | **Control** | **Summary measure, p-value** | **Direction of effect** |
| --- | --- | --- | --- | --- |
| Brown 2002, UK^23^  n=61  PIFU n=30  Control n=31 | Median (IQR) (raw scores)  No summary scores  **Baseline, 12 months**  Body image:5(2), 5(2.25)  Sexual functioning: 3(5.5), 2(4)  Arm symptoms: 3(0), 3(1)  Breast symptoms:4(1), 4(1)  Systematic therapy side effects: 9(2.25), 9(3.25)  Future perspective: 1.5(1), 1(1) | Median (IQR) (raw scores)  No summary scores  **Baseline, 12 months**  Body image: 5(2), 5(2)  Sexual functioning:3(4), 2(1.5)  Arm symptoms:3.5(2), 4(2)  Breast symptoms:5(2), 5(2)  Systematic therapy side effects:10(2.5), 9(2.5)  Future perspective:2(0), 2(0) | No summary measures stated.  P=0.003 (arm symptoms score baseline)  P=0.028 (arm symptoms score 6 months)  P=0.033 (breast symptoms score baseline)  P=0.024 (breast symptoms score 12 months) | Arm symptom scores higher (better) in routine FU at baseline and 6 months.  Breast symptoms scores higher (better) in routine FU at 6 and 12 months.  No significant differences between groups for other sub-scales.  *NB 6 month results also presented, not extracted.* |
| Kirshbaum 2017, Australia & UK^25^  n=112  PIFU n=56  Control n=56 | Mean (SD) of transformed score+  No summary scores  Baseline, 24 months  Body image:78.7 (21.1), 79.4 (25.9)  Sexual functioning: 28.0 (26.2), 24.7 (25.0)  Sexual enjoyment:77.8 (27.2), 61.1 (25.1)  Arm symptoms:14.5 (14.3), 12.0 (15.0)  Breast symptoms:23.2 (20.2), 11.5 (12.8)  Systematic therapy side effects: 26.0 (22.1), 26.4 (20.7)  Future perspective:61.5 (24.1), 55.2 (30.1) | Mean (SD) of transformed score+  No summary scores  Baseline, 24 months  Body image:75.7 (28.6), 80.4 (26.2)  Sexual functioning: 27.6 (29.0), 18.4 (23.7)  Sexual enjoyment:72.7 (25.0), 72.7 (32.7)  Arm symptoms:13.3 (18.2), 15.4 (20.8)  Breast symptoms:20.9 (18.3), 13.4 (16.8)  Systematic therapy side effects: 28.9 (21.1), 25.5 (22.4)  Future perspective:65.7 (30.3), 58.3 (31.2) | No summary measure or p-values reported. | Type of FU not found to be a statistically significant predictor on multilevel linear regression modelling.  *NB 6, 12 and 18 month results also presented (not extracted)* |
| Riis 2020, Denmark^27^  n=129  PIFU n=62  Control n=67 | Not stated | Not stated | No summary measure or p-values reported. | No significant differences between groups (for any sub-scale or symptom). |

*** *European Organisation for Research and Treatment of Cancer (EORTC) QLQ-BR23; 23 item questionnaire; breast-cancer specific QoL domains: body image, sexual functioning, future perspectives, physical symptoms and side effects of treatment; score range for all items 0-100; higher score on functional scales indicates better level of functioning; high score for symptom scales indicates higher level of symptoms/problems +based on multilevel linear regression modelling*

**FACT-G questionnaire***

| **Study** | **PIFU** | **Control** | **Summary measure, p-value** | **Direction of effect** |
| --- | --- | --- | --- | --- |
| Sheppard 2009, UK^26^  n=237  PIFU n=107*  Control n=107*  *only number analysed by group | Mean (SD)  FACT-G  Baseline: 81.6 (15.2)  9 months (adjusted):83.3 (no SD)  18 months (adjusted): 81.4 (no SD)  Breast sub-scale  Baseline: 25.1 (6.0)  9 months (adjusted):21.7 (no SD)  18 months (adjusted): 20.1 (no SD)  Endocrine sub-scale  Baseline: 54.9 (11.2)  9 months (adjusted):56.5 (no SD)  18 months (adjusted): 57.4 (no SD)  FACT-B + ES  Baseline: 161.7 (26.0)  9 months (adjusted):162.2 (no SD)  18 months (adjusted):158.9 (no SD) | Mean (SD)  FACT-G  Baseline: 82 (18.2)  9 months (adjusted):82.9 (no SD)  18 months (adjusted): 81.3 (no SD)  Breast sub-scale  Baseline: 24.4 (5.5)  9 months (adjusted):23.2 (no SD)  18 months (adjusted): 21.8 (no SD)  Endocrine sub-scale  Baseline: 55.8 (9.8)  9 months (adjusted):56.4 (no SD)  18 months (adjusted): 58.7 (no SD)  FACT-B + ES  Baseline: 162.2 (26.0)  9 months (adjusted):162 (no SD)  18 months (adjusted): 161.9 (no SD) | Adjusted mean 18 months PIFU-control (95% CI):  FACT-G:  0.1 (-3.2 to 3.4), p=0.952  Breast sub-scale:  -1.7 (-3.2 to 0.5)  p=0.058  Endocrine sub-scale:  -1.2 (-4.2 to 1.6), p=0.388  FACT-B + ES:  -1.6 (-4.6 to 8.0), p not stated. | FACT-G: no significant difference  at 18 months.  Breast sub-scale: no significant difference at 18 months; approaching significance in favour of PIFU.  Endocrine sub-scale: no significant difference at 18 months.  FACT-B + ES: no significant difference at 18 months. |

** Functional Assessment of Cancer Therapy (FACT) questionnaire; 27-item questionnaire measuring four domains of HRQOL in cancer patients: physical, social, emotional, and functional well-being; extensions include additional breast and endocrine sub-scales. Higher scores indicate better QoL.*

**Hospital Anxiety and Depression scale (HADS)***

| **Study** | **PIFU** | **Control** | **Summary measure, p-value** | **Direction of effect** |
| --- | --- | --- | --- | --- |
| Brown 2002, UK^23^  n=61  PIFU n=30  Control n=31 | *Anxiety*  Median (range)  Baseline:3.5 (0-15)  6 months: 4 (0-10)  12 months: 4 (0-12)  1 probable case, 6 borderline. | *Anxiety*  Median (range)  Baseline:5 (0-15)  6 months:5 (0-18)  12 months:6.5 (0-16)  3 probable cases, 6 borderline. | No summary measure.  P values >0.05 at all timepoints. | Slightly higher values in control group, but no significant differences at baseline or 12 months, or over time between the groups. |
|  | *Depression*  Median (range)  Baseline: 1 (0-13)  6 months: 1.5 (0-7)  12 months: 1 (0-7)  1 probably case. | *Depression*  Median (range)  Baseline:2 (0-9)  6 months:2 (0-7)  12 months: 2 (0-8)  1 borderline case. | No summary measure.  P values >0.05 at all timepoints. | Slightly higher values in control group, but no significant differences at baseline or 12 months, or over time between the groups. |
| Kirshbaum 2017, Australia & UK^25^  n=112  PIFU n=56  Control n=56 | *Anxiety*  Mean (SD)  Baseline: 6.59 (3.42)  6 months: 6.39 (4.05)  12 months: 6.32 (3.72)  18 months: 7.01 (4.21)  24 months: 7.00 (4.60) | *Anxiety*  Mean (SD)  Baseline: 5.71 (4.43)  6 months: 5.53 (4.12)  12 months: 5.47 (4.80)  18 months: 5.83 (4.52)  24 months: 5.74 (5.00) | No summary measure or p-values reported. | Type of FU not found to be a statistically significant predictor on multilevel linear regression modelling. |
|  | *Depression*  Mean (SD)  Baseline: 3.19 (2.72)  6 months: 2.99 (2.89)  12 months: 3.44 (3.09)  18 months: 3.73 (3.39)  24 months: 3.71 (3.24) | *Depression*  Mean (SD)  Baseline: 3.53 (3.45)  6 months: 3.58 (3.70)  12 months: 3.59 (4.03)  18 months: 3.46 (3.18)  24 months: 3.94 (4.12) | No summary measure or p-values reported. | Type of FU not found to be a statistically significant predictor on multilevel linear regression modelling. |
| Koinberg 2004, Sweden^7^  n=264  PIFU n=133  Control n=131 | *Anxiety*  6 months: 9.7%  12 months:8.0%  24 months:9.6%  60 months:11.6% | *Anxiety*  6 months:5.6%  12 months:7.0%  24 months:4.4%  60 months:6.7% | Relative risk (RR), 95% CI (no p-values reported):  6 months:1.8 (0.7, 4.8)  12 months:1.2 (0.4, 3.1)  24 months:2.3 (0.8, 6.9)  60 months:1.8 (0.6, 5.1) | Slightly higher % of anxiety in PIFU group compared with routine clinic follow-up, but no statistically significant differences between groups. |
|  | *Depression*  Those with score 11-20 classified as cases.  6 months:0.8%  12 months:0.9%  24 months:1.8%  60 months:5.2% | *Depression*  Those with score 11-20 classified as cases.  6 months:0.8%  12 months:1.7%  24 months:1.8%  60 months:3.2% | Relative risk (RR), 95% CI (no p-values reported):  6 months:1.0 (0.6, 16.4)  12 months:0.5 (0, 5.8)  24 months:1.0 (0.1, 7.2)  60 months:1.7 (0.4, 7.2) | Slightly higher % of depression in PIFU group (at 60 months), but no statistically significant differences between groups. |

** Hospital Anxiety and Depression scale (HADS): 14 items on 2 sub-scales for anxiety and depression, each scored 0-3; highest possible score on sub-scale is 21; scores of 7 or less indicate non-cases, scores of 8–10 indicate borderline cases, scores of over 11 indicate the presence of probable anxiety or depression.*

**GHQ12 questionnaire***

| **Study** | **PIFU** | **Control** | **Summary measure, p-value** | **Direction of effect** |
| --- | --- | --- | --- | --- |
| Sheppard 2009, UK^26^  n=237  PIFU n=107*  Control n=107*  *only number analysed by group | Mean (SD)  Baseline: 22.9 (4.8)  9 months (adjusted):23.0 (no SD)  18 months (adjusted): 22.8 (no SD)  Cases (n (%)):  Baseline: 17 (16%)  9 months: 23 (22%)  18 months: 15 (14%) | Mean (SD)  Baseline:23.2 (no SD)  9 months (adjusted):22.4 (no SD)  18 months (adjusted): 23.0 (no SD)  Cases (n (%)):  Baseline:16 (15%)  9 months: 13 (12%)  18 months:21 (20%) | Adjusted mean 18 months PIFU-control (95% CI):-0.1 (-1.4, 1.0), p=0.767  Not stated for cases. | No significant differences between groups at 18 months (adjusted mean difference). Also little change over time. |

*** *General Health Questionnaire (GHQ12) for psychological morbidity; 12 questions score 1-4, maximum score 48; responses score as positive or negative using official GHQ scoring mechanism; individuals with 4 or more positive responses were considered ‘cases’ (i.e. indicative of having poor psychological health).*

**Contact with HCPs/resource use**

| **Study** | **PIFU** | **Control** | **Summary measure, p-value** | **Direction of effect** |
| --- | --- | --- | --- | --- |
| Brown 2002, UK^23^  n=61  PIFU n=30  Control n=31 | Telephone call to breast care nurse: 2  Referral to hospital by GP:4  Two participants (6.7%) requested an outpatient FU appointment at the breast clinic on completion of the study | Telephone call to breast care nurse:1  Referral to hospital by GP:3 | No summary measure or p-values reported. | Similar contact with HCP in both groups (low numbers of contacts). |
| Gulliford 1997, UK^24^  n=196  PIFU n=97*  Control n=96*  * missing data for n=3 | Telephone calls: 8  Cancer related GP visits: 4  Non cancer related GP visits: 49 | Telephone calls: 11  Cancer related GP visits: 7  Non cancer related GP visits: 39 | No summary measure or p-values reported. | Similar contact via the telephone advice line or the GP in both arms (low numbers). |
| Koinberg 2004, Sweden^7^  n=264  PIFU n=133  Control n=131 | Number of patients with the specified contact, total number of contacts, events/person-year  Physician visits: 131, 459, 0.96  Nurse visits: 131, 104, 0.22  Telephone contacts:131, 268, 0.56  Other consultations:76, 192, 0.67  Mammography:133, 388, 0.80  Pulmonary x-ray:131, 98, 0.2  Scintigraphy, CT scans:131, 84, 0.18  US, other imaging:131, 26, 0.05  Laboratory evaluations:131, 299, 0.63  Cytologies, biopsies:131, 17, 0.04 | Number of patients with the specified contact, total number of contacts, events/person-year  Physician visits: 131, 902, 1.62  Nurse visits: 131, 16, 0.03  Telephone contacts:131, 91, 0.16  Other consultations:77, 163, 0.47  Mammography:131, 368, 0.66  Pulmonary x-ray:110, 38, 0.08  Scintigraphy, CT scans:109, 65, 0.14  US, other imaging:109, 20, 0.04  Laboratory evaluations:130, 235, 0.43  Cytologies, biopsies:109, 16, 0.04 | No summary measure reported.  P=0.01  P<0.01  P<0.01  P=0.30  P<0.01  P=0.07  P=0.82  P=0.80  P=0.73  P=0.1 | Statistically significant difference in number of physician visits, nurse visits and telephone contacts.  No significant differences in number of laboratory and imaging evaluations, except mammography (significantly higher rate in PIFU group). |
| Sheppard 2009, UK^26^  n=237  PIFU n=107*  Control n=107*  *only number analysed by group | Contacts with specialist nurses:61  (rate of 0.38 contacts per person-year) | Contacts with specialist nurses (in addition to routine review):68  (rate of 0.42 contacts per person-year) | No summary measure or p-values reported. | Similar amount of contact in both groups. |
| Riis 2020, Denmark^27^  n=129  PIFU n=62  Control n=67 | Mean (95% CI) number of clinician consultations attended during study period: 2.1 (1.6, 2.6) | Mean (95% CI) number of clinician consultations attended during study period: 4.3 (3.9, 4.7) | No summary measure reported. P<0.001 | Significantly fewer clinician consultations in PIFU arm. |

**Satisfaction and accessibility scale (SaaC)***

| **Study** | **PIFU** | **Control** | **Summary measure, p-value** | **Direction of effect** |
| --- | --- | --- | --- | --- |
| Koinberg 2004, Sweden^7^  n=264  PIFU n=133  Control n=131 | Dichotomised for satisfaction  versus dissatisfaction outcomes (%).  Accessibility by phone (satisfaction)  6 months:96.3  18 months:100  24 months:99  60 months:96.5  Accessibility to medical centre (satisfaction)  6 months:96.5  18 months:98.9  24 months:97.0  60 months:97.7  Satisfaction with medical centre:  6 months:97.6  18 months:96.4  24 months:92.3  60 months:91.6 | Dichotomised for satisfaction  versus dissatisfaction outcomes (%).  Accessibility by phone (satisfaction)  6 months:96.3  18 months:100  24 months:99  60 months:100  Accessibility to medical centre (satisfaction)  6 months:97.5  18 months:100  24 months:100  60 months:100  Satisfaction with medical centre:  6 months:98.4  18 months:100  24 months:97.4  60 months:98.9 | Relative risk (RR), 95% CI (no p-values reported):  Accessibility by phone  6 months:1.0 (0.2, 4.1)  18 months:1.0 (N/A)  24 months:0.9 (0.6, 15.3)  60 months:1.0 (0.9, 1.0)  Accessibility to medical centre  6 months:0.7 (0.1, 3.1)  18 months:1.0 (0.9, 1.0)  24 months: 1.0 (0.9, 1.0)  60 months: 1.0 (0.9, 1.0)  Satisfaction with medical centre:  6 months:0.6 (0.1, 3.9)  18 months:1.0 (1.0, 1.0)  24 months:0.3 (0, 1.2)  60 months:0.1 (0.0, 0.9) | Slightly less satisfaction in PIFU arm with medical centre at 24 and 60 months, but no statistically significant differences between groups at any time point except for the ‘satisfaction with medical centre’ item at 60 months (less satisfaction in PIFU arm). |

** Satisfaction and accessibility scale (SaaC) designed specifically for study; 9 questions about patient satisfaction with FU system and accessibility of breast medical services including whether or not the patient wanted to change FU routines; 4 point scale ranging from ‘‘very satisfied’’ to ‘‘very dissatisfied’’. Higher scores indicates greater level of satisfaction/accessibility.*

**Four items** **from Patient Experience Questionnaire (PEQ)***

| **Study** | **PIFU** | **Control** | **Summary measure, p-value** | **Direction of effect** |
| --- | --- | --- | --- | --- |
| Riis 2020, Denmark^27^  n=129  PIFU n=62  Control n=67 | Proportion satisfied or very satisfied with FU:>88% at each time point  *Estimated from graph:*  Proportion that wished for procedures that were not offered: 1-7% (depending on time-point)  Proportion that had a concern that did not lead to contact with the department: 5-17% (depending on time-point)  Proportion that would have liked more information on something: 4-18% (depending on time-point) | Proportion satisfied or very satisfied with FU:>88% at each time-point  *Estimated from graph:*  Proportion that wished for procedures that were not offered: 3-14% (depending on time-point)  Proportion that had a concern that did not lead to contact with the department: (depending on time-point): 7-12%  Proportion that would have liked more information on something:3-20% (depending on time-point) | No summary measure or p-values reported. | Similar proportions in both groups. |

** Patient Experience Questionnaire (PEQ): satisfaction measured by a single item with a 5-point scale (1=very unsatisfied, 5=very satisfied); three questions addressed unmet needs toward (1)* *procedures not offered, (2) concerns not provided for and (3) missing information.*

| **Study** | **PIFU** | **Control** | **Summary measure, p-value** | **Direction of effect** |
| --- | --- | --- | --- | --- |
| Gulliford 1997, UK^24^  n=196  PIFU n=97*  Control n=96*  * missing data for n=3 | n(%)  Clinic visit  Was reassuring: 69 (88%)  Wish to continue with hospital based follow up rather than with general practitioner alone:70 (89%)  Future visit schedule  Prefer more frequent:10 (16%)  Prefer less frequent:22 (35%) | n(%)  Clinic visit  Was reassuring: 73 (94%)  Wish to continue with hospital based follow up rather than with general practitioner alone: 74 (94%)  Future visit schedule  Prefer more frequent:7 (11%)  Prefer less frequent:17 (25%) | No summary measure or p-values reported. | Similar proportions in both arms in terms of preferences. |

**Feelings about clinic visits and desired future frequency***

** Questionnaires based on the Medical Research Council quality of life questionnaire were used and included additional questions relating to frequency of follow up in hospital and general practice, satisfaction with clinic visits, and the time and expense entailed in clinic attendance.*

**Fear of recurrence***

| **Study** | **PIFU** | **Control** | **Summary measure, p-value** | **Direction of effect** |
| --- | --- | --- | --- | --- |
| Sheppard 2009, UK^26^  n=237  PIFU n=107*  Control n=107*  *only number analysed by group | Mean (SD)  Baseline: 5.8 (3.1)  9 months (adjusted):5.6 (no SD)  18 months (adjusted): 5.6 (no SD) | Mean (SD)  Baseline: 6.0 (2.9)  9 months (adjusted):5.7 (no SD)  18 months (adjusted): 5.0 (no SD) | Adjusted mean 18 months PIFU-control (95% CI):  0.5 (-0.3, 1.0), p=0.066 | Slightly higher levels of fear in PIFU group, but not statistically significant. |

*** *Fear of recurrence measured by 3-item questionnaire designed by author of FACT-G (not sufficiently tested at the time of study); higher score indicates greater level of fear.*

**Isolation***

| **Study** | **PIFU** | **Control** | **Summary measure, p-value** | **Direction of effect** |
| --- | --- | --- | --- | --- |
| Sheppard 2009, UK^26^  n=237  PIFU n=107*  Control n=107*  *only number analysed by group | 14/97 (14%) patients stated they had felt isolated | 9/99 (9%) patients stated they had felt isolated | No summary measure. P=0.245 | Slightly higher levels of isolation in the PIFU group, but not statistically significant. |

*** *measured by asking the patients in both groups to record at 9 and 18 months whether they had felt isolated since their last review.*

**Adherence to treatment***

| **Study** | **PIFU** | **Control** | **Summary measure, p-value** | **Direction of effect** |
| --- | --- | --- | --- | --- |
| Riis, 2020, Denmark^27^  n=129  PIFU n=62  Control n=67 | 51/59 (86%) adherent to endocrine treatment at end of study  23% changed endocrine treatment during study period | 55/60 (92%) adherent to endocrine treatment at end of study  28% changed endocrine treatment during study period | No summary measures. P=1.0  P=0.56 | No significant difference between groups. |

** Adherence to treatment categorised as (1) adherence to the primary prescribed drug during the whole study period, (2) change in endocrine therapy during the study period with continued adherence, or (3) discontinuation of adjuvant therapy.*

***Endometrial cancer*** ***(Jeppesen 2018)^30^***

n=212, PIFU n=105, control n=107

| **Outcome** | **PIFU** | **Control** | **Summary measure, p-value** | **Direction of effect** |
| --- | --- | --- | --- | --- |
| Fear of Cancer Recurrence Inventory (FCRI)*  Mean (SD) | 1 month: 44.8 27.3  3 months: 43.8 28.5  6 months: 42.8 26.9  10 months: 43.4 26.5 | 1 month: 48.5 (28.8)  3 months: 41.9 (28.4)  6 months: 40.4 (29.5)  10 months: 40.5 (28.5) | Estimated difference in total FCRI score (95% CI), based on linear regression analysis adjusted for hospital and baseline score:  -5.9 (-10.9; -0.9), p= 0.02 | Greater reduction on FCR in the control group compared with PIFU (statistically significant difference). |
| FCRI % with clinical FCR^+^ | 1 month: 16.5  3 months: 22.8  6 months: 20.3  10 months: 17.7 | 1 month: 26.0  3 months: 24.7  6 months: 24.7  10 months: 22.1 | Odds ratio (95% CI) from logistic regression analysis adjusted for hospital and baseline score:  0.9 (0.32;2.67), p= 0.89 | No statistically significant difference between groups. |
| Contact with HCPs within 10 months.  Median (IQR), total number | | | | |
| Cancer-related visits to GP | 1 (0-4), 213 | 1 (0-3), 135 | No summary measure. P=0.77 | No statistically significant difference between groups. |
| Cancer-related visits to privately-practising gynaecologist | 0 (0-0), 17 | 0 (0-0), 10 | No summary measure. P=0.31 |  |
| Telephone contacts with the departments of gynaecology | 0 (0-0), 20 | 0 (0-0), 7 | No summary measure. P=0.15 |  |
| Examinations performed at the departments of gynaecology | 0 (0-0), 19 | 2 (1-2), 139 | No summary measure. P<0.01 | Significantly fewer hospital examinations in PIFU group (primary scheduled due to symptoms). |

** Fear of Cancer Recurrence Inventory (FCRI) includes seven subscales (triggers, severity, psychological distress, reassurance-seeking, coping strategies, functioning impairments and insight); score 0-168; higher score indicates higher level of FCR; + cut-off value for clinical FCR defined as score of ≥ 16 on the Severity subscale (range 0–36)*

***Colorectal cancer***

| **Outcome** | **PIFU** | **Control** | **Summary measure, p-value** | **Direction of effect** |
| --- | --- | --- | --- | --- |
| **Ohlsson 1995^28^** n=107, PIFU n=54, control=53 | | | | |
| Tumour recurrence (n patients) | 18 (33.3%) | 17 (32.1%) | No summary measure or p-values reported. | Slightly poorer outcomes in PIFU group for all outcome measures, but no statistically significant differences (where stated). |
| Median (range) time (years) to first recurrence | 2.0 (0.8-5.6) | 1.7 (0.3-7.6) | No summary measure, p>0.05. |  |
| Re-resection (attempted curative reoperation) | 3 | 5 (4 with asymptomatic recurrence) | No summary measure or p-values reported. |  |
| Mortality (end of study) | 22/54 (41%) | 15/53 (28%) | No summary measure or p-values reported. |  |
| Five-year survival rate | 67% | 75% | No summary measure, p>0.05. |  |
| Cancer-specific five-year survival rate | 71% | 78% | No summary measure, p>0.05. |  |
| Median survival after recurrence | 2.7 | 3.5 | No summary measure, p>0.05. |  |
| Five-year survival after recurrence | 22% | 29% | No summary measure or p-values reported. |  |
| Adherence | 19/54 (35%) did not return at all after randomisation; 3 wanted a control examination | 1/53 (2%) did not want FU after two years | No summary measure or p-values reported. | 35% in the PIFU arm did not have contact with the hospital after surgery (either to submit faecal samples or because of symptoms). 98% of patients adhered to the scheduled FU. |
| **Hovdenak-Jacobsen 2021^8^** n=336, PIFU n= 168, control=168 | | | | |
| No. of outpatient clinical visits-doctor | 342 | 521 | No summary measure, p<0.001. | Significantly more doctor visits in routine FU arm. |
| No. of extra clinical visit (not routine) | 144 | 148 | No summary measure, p=0.20. | No difference |
| No. of clinical visits initiated by patients | 132 | 144 | No summary measure, p=0.33. | No difference |
| No. of outpatient clinical visits-nurse | 358 | 340 | No summary measure, p=0.27. | No difference |
| No. of telephone consultations | 99 | 111 | No summary measure, p=0.59. | No difference |
| Number of any contacts ≥15 | 17% | 7% | No summary measure, p=0.004. | Significant difference in pattern of contact. |
| Compliance with PIFU protocol | 64/168 (38.1%) patients had planned routine clinical visits due to clinical decisions or because of the hospital’s non-compliance with the protocol. | 25/168 (14.9%) patients had no planned routine visit during the first year of follow-up. | No summary measure or p-value reported. | Greater number of protocol violations in PFU arm compared with routine FU arm. |
| Patient involvement in FU (numerical rating scale from 1-10), 6 items | Median score higher in PIFU arm for 5/6 items; statistically significant for 2 of these *(“health professionals have strived to understand what has been most important to me”, “privacy and quietness have characterised my conversations with health professionals”).* Other items were*: “I had the opportunity to talk with health professionals regarding my questions and concerns”; “I experienced presence and attention in consultations with health professionals”; “I was taken on board when decisions were made”; The health professionals have shown interest in my preferences for involving my next of kin in the follow-up.”* | | | |
| Patient-perceived satisfaction (information, help and support). *Items scored on a 5-point Likert scale. Item 1-3 were dichotomised into ‘sufficient/almost sufficient’ including the three*  *most positive categories vs ’not sufficient/no information’ including the two remaining categories. For items 4-5, the category ‘No help needed’ was omitted from analysis, and the remaining four options were dichotomised into ’high/some degree’ vs ’insufficient/no help’.* | Statistically significant greater percentage of satisfaction for all 5 items in favour of PIFU.  Items were: *“How would you describe the information you received at the hospital regarding crucial symptoms that*  *require your action?”; “How would you describe the information you received at the hospital regarding where to make contact, in case of concerns and symptoms?”; “How would you describe the information you received at the hospital regarding potential late effects from your disease and treatment?”; “Have you received adequate help and support during your follow-up, related to physical problems (i.e. pain and fatigue)?”; “Have you received adequate help and support during your follow-up, related to emotional problems (i.e. fear of cancer recurrence and depressive thoughts)?”* | | | |
| **Hovdenak-Jacobsen 2022^29^**  Functional Assessment of Cancer Therapy – colorectal (FACT-C) and FACT-C/Treatment Outcome Index (TOI)*  *Reported in conference abstract* | Not reported | Not reported | No summary measure or p-value reported. | No statistically significant differences between groups. |

***** *Functional Assessment of Cancer Therapy – colorectal (FACT-C): 37 item colorectal specific scale with 5 subscales: physical well-being, social/family well-being, emotional well-being, functional well-being, colorectal cancer subscale (CCS); each item rated 0-4, higher score indicates better QoL. FACT-C/Treatment Outcome Index (TOI)* *sums the FACT-C physical and functional domains and the CCS.*

***Melanoma (Ackermann 2022)^31^***

n=100, PIFU n=51, control=49

| **Outcome** | **PIFU + usual care** | **Usual care** | **Summary measure^a^, p-value** | **Direction of effect** |
| --- | --- | --- | --- | --- |
| No of clinic visits (median, IQR) | 2 (0-9) | 1 (0-6) | RR 1.5 (95% CI 1.1 -2.1) | Significantly more clinic visits in PIFU + usual care arm. |
| No. of skin lesions surgically excised (median, IQR) | 1 (0-5) | 0 (0-10) | RR 1.1 (95% CI 0.6-2.0) | Both arms equally likely to have lesions surgically excised. |
| Participants with ≥1 surgical excision of skin lesion | 21 (41%) | 28 (57%) | Difference in proportions (95% CI): 16 (-3 to 35) | No significant difference |
| Participants with new keratinocyte cancer diagnoses | 12 (25%) | 11 (22%) | Difference in proportions (95% CI): 1 (-18 to 15) | No significant difference |
| Participants with new or recurrent melanoma diagnoses | 8 (16%) | 3 (6%) | Difference in proportions (95% CI): 10 (-2 to 23) | No significant difference |
| Participants with recurrent melanoma | 1 (2%) | 0 (0%) | Not reported | Not reported |
| New melanoma diagnoses prompted by visit type | Unscheduled visit:5 (10%)  Scheduled visit: 3 (6%) | Unscheduled visit:0 (0%)  Scheduled visit:3 (6%) | Difference in proportions (95% CI)  Unscheduled visit:10 (2-19)  Scheduled visit:0 (-9 to 10) | Significantly more unscheduled visits in PIFU+ usual care arm |
| Fear of Cancer Recurrence Inventory severity subscale* | Mean (SD) 14.6 (5.1) | Mean (SD) 15.9 (6.0) | Between-group  mean score difference −1.3 (95% CI, −3.1 to 0.5) | No significant difference |
| Change in total Depression Anxiety and Stress Scale+ | Mean (SD) 6.5 (7.1) | Mean (SD) 9.9 (14.5) | Between-group  mean score difference −1.4 (95% CI, −5.8 to 2.0) | No significant difference |

** Fear of Cancer Recurrence Inventory (FCRI) includes seven subscales (triggers, severity, psychological distress, reassurance-seeking, coping strategies, functioning impairments and insight); score 0-168; higher score indicates higher level of FCR; cut-off value for clinical FCR defined as score of ≥ 16 on the Severity subscale (range 0–36); + Depression Anxiety Stress Scales-21 (DASS-21), 21 items (7 items on each sub-scale scored 0-3), higher scores indicate greater depression/anxiety/stress; ^a^ effect of the intervention estimated through logistic regression for binary outcomes, multiple linear regression for continuous outcomes and Poisson regression for count outcomes; negative binomial regression used for count outcomes where overdispersion was present; for non-continuous outcomes, effect of intervention at 6 months estimated using univariable methods; for continuous outcomes, baseline measurement of the outcome included in model as covariate; for new melanoma diagnoses, difference in proportions and confidence intervals calculated using the χ2 method without continuity correction*

7 Koinberg IL, Fridlund B, Engholm GB, Holmberg L. Nurse-led follow-up on demand or by a physician after breast cancer surgery: a randomised study. Eur J Oncol Nurs. 2004;8(2):109-17; discussion 18-20.

8 Hovdenak Jakobsen I, Vind Thaysen H, Laurberg S, Johansen C, Juul T, Group FS. Patient-led follow-up reduces outpatient doctor visits and improves patient satisfaction. One-year analysis of secondary outcomes in the randomised trial Follow-Up after Rectal CAncer (FURCA). Acta Oncol. 2021;60(9):1130-9.

23 Brown L, Payne S, Royle G. Patient initiated follow up of breast cancer. Psychooncology. 2002;11(4):346-55.

24 Gulliford T, Opomu M, Wilson E, Hanham I, Epstein R. Popularity of less frequent follow up for breast cancer in randomised study: initial findings from the hotline study. BMJ. 1997;314(7075):174-7.

25 Kirshbaum MN, Dent J, Stephenson J, Topping AE, Allinson V, McCoy M, et al. Open access follow-up care for early breast cancer: a randomised controlled quality of life analysis. Eur J Cancer Care (Engl). 2017;26(4).

26 Sheppard C, Higgins B, Wise M, Yiangou C, Dubois D, Kilburn S. Breast cancer follow up: a randomised controlled trial comparing point of need access versus routine 6-monthly clinical review. Eur J Oncol Nurs. 2009;13(1):2-8.

27 Riis CL, Jensen PT, Bechmann T, Moller S, Coulter A, Steffensen KD. Satisfaction with care and adherence to treatment when using patient reported outcomes to individualize follow-up care for women with early breast cancer - a pilot randomized controlled trial. Acta Oncol. 2020;59(4):444-52.

28 Ohlsson B, Breland U, Ekberg H, Graffner H, Tranberg KG. Follow-up after curative surgery for colorectal carcinoma. Randomized comparison with no follow-up. Dis Colon Rectum. 1995;38(6):619-26.

29 Hovdenak I, Juul T, Thomsen Bernstein I, Christensen P, Hjerrild Iversen L, Johansen C, et al., editors. The effect of a patient-led follow-up program after rectal cancer surgery on symptom burden and quality of life -a randomised controlled trial2022.

30 Jeppesen MM, Jensen PT, Hansen DG, Christensen RD, Mogensen O. Patient-initiated follow up affects fear of recurrence and healthcare use: a randomised trial in early-stage endometrial cancer. BJOG. 2018;125(13):1705-14.

31 Ackermann DM, Dieng M, Medcalf E, Jenkins MC, van Kemenade CH, Janda M, et al. Assessing the Potential for Patient-led Surveillance After Treatment of Localized Melanoma (MEL-SELF): A Pilot Randomized Clinical Trial. JAMA Dermatol. 2022;158(1):33-42.
