# Supplementary material for: A systematic review of the effectiveness of patient‐initiated follow‐up after cancer
Source: Cancer Med. 2023 Aug 21;12(18):19057–71. doi: 10.1002/cam4.6462 (PMC10557867; doi:10.1002/cam4.6462)
Supplement: Supplementary file 1 — Data S1 [file CAM4-12-19057-s001.zip › cam46462-sup-0001-Supinfo/Suppl 5 Excluded studies.docx]

**Excluded studies**

| *Study* | *Reason* |
| --- | --- |
| De Zoysa N, Lee A, Joshi A, Guerrero-Urbano T, Lei M, McGurk, M, Lyons A, Cascarini L, Jeannon J, Simo RS, Ali S, Oakley R. Developing a follow-up surveillance protocol in head and neck oncological surgery: enhanced ‘traffic light’ surveillance – a prospective feasibility study. Clinical Otolaryngology 2015; 42: 439–494 | Prospective controlled but not randomised. |
| Hovdenak Jakobsen I, Juul T, Thaysen HV, Johansen C, Laurberg S. Differences in baseline characteristics and 1-year psychological factors between participants and non-participants in the randomized, controlled trial regarding patient-led follow-up after rectal cancer (FURCA). Acta Oncol. 2019 May;58(5):627-633. doi: 10.1080/0284186X.2019.1581948. Epub 2019 Mar 5. PMID: 30836806. | Not reporting differences between randomised arms. |
| Kjeldsen BJ, Kronborg O, Fenger C, Jørgensen OD. A prospective randomized study of follow-up after radical surgery for colorectal cancer. Br J Surg. 1997 May;84(5):666-9. PMID: 9171758. | Not PIFU. Intensive vs less intensive FU (longer time intervals). |
| Kokko R, Hakama M, Holli K. Follow-up cost of breast cancer patients with localized disease after primary treatment: a randomized trial. Breast Cancer Res Treat. 2005 Oct;93(3):255-60. doi: 10.1007/s10549-005-5199-2. PMID: 16132530. | Not PIFU. Arms differed in frequency of follow-up visits (every third or sixth month) and in intensity of diagnostic examinations. |
| Mant D, Gray A, Pugh S, Campbell H, George S, Fuller A, et al. A randomised controlled trial to assess the cost-effectiveness of intensive versus no scheduled follow-up in patients who have undergone resection for colorectal cancer with curative intent. Health Technol Assess 2017;21(32). | Not PIFU. Augmenting symptomatic follow-up in primary care with two intensive methods of follow-up: monitoring of a tumour marker in primary care and intensive imaging in hospital. |
| Moore S, Corner J, Haviland J, Wells M, Salmon E, Normand C, Brada M, O'Brien M, Smith I. Nurse led follow up and conventional medical follow up in management of patients with lung cancer: randomised trial. BMJ. 2002 Nov 16;325(7373):1145. doi: 10.1136/bmj.325.7373.1145. Erratum in: BMJ 2002 Dec 14;325(7377):1386. PMID: 12433764; PMCID: PMC133453. | Not PIFU. Nurse led arm had regular appointments. |
| Secco GB, Fardelli R, Gianquinto D, Bonfante P, Baldi E, Ravera G, Derchi L, Ferraris R. Efficacy and cost of risk-adapted follow-up in patients after colorectal cancer surgery: a prospective, randomized and controlled trial. Eur J Surg Oncol. 2002 Jun;28(4):418-23. doi: 10.1053/ejso.2001.1250. PMID: 12099653. | Not PIFU. Intensive FU for high-risk pts versus less intensive FU for low-risk pts. |
| van der Hout A, van Uden-Kraan CF, Holtmaat K, Jansen F, Lissenberg-Witte BI, Nieuwenhuijzen GAP, Hardillo JA, Baatenburg de Jong RJ, Tiren-Verbeet NL, Sommeijer DW, de Heer K, Schaar CG, Sedee RE, Bosscha K, van den Brekel MWM, Petersen JF, Westerman M, Honings J, Takes RP, Houtenbos I, van den Broek WT, de Bree R, Jansen P, Eerenstein SEJ, Leemans CR, Zijlstra JM, Cuijpers P, van de Poll-Franse LV, Verdonck-de Leeuw IM. Role of eHealth application Oncokompas in supporting self-management of symptoms and health-related quality of life in cancer survivors: a randomised, controlled trial. Lancet Oncol. 2020 Jan;21(1):80-94. | Not PIFU. Evaluation of eHealth  self-management application, which aims to support survivors in self-management. |
